# ^13^C longitudinal relaxation time measurements and DFT-GIAO NMR computations for two ammonium ions of a tetraazamacrocyclic *scorpiand* system

**DOI:** 10.1007/s10847-013-0298-x

**Published:** 2013-03-02

**Authors:** Ryszard B. Nazarski

**Affiliations:** Laboratory of Molecular Spectroscopy, Faculty of Chemistry, University of Łódź, ul. Tamka 12, 91-403 Łódź, Poland

**Keywords:** **‘**Wrong-way’ protonation shift, Amino-pendant cyclams, NMR pH-titration, Protonated polyamines, Nitrate receptors, Dipolar relaxation, OPLS-AA force field, DFT-D3 dispersion correction

## Abstract

**Abstract:**

Spin–lattice relaxation times, *T*_1_s, for ^13^C nuclei in two cations H_*n*_ **1** ^*n*+^ (*n* = 1, 5) of *N*-(2-aminoethyl)-cyclam (**1**, *scorpiand*) were determined by means of ^13^C{^1^H} NMR experiments in aqueous solution at pH 11.5 and 0.2. The theoretical study [modeling with OPLS-AA, B3LYP/6-31G(*d*) geometry optimizations, dispersion-corrected energies (DFT-D3), and DFT-GIAO predictions of the NMR chemical shifts (including an IEF-PCM simulation of hydration)] was also done for several conformers of the tautomer *iso*-H_4_ **1** ^4+^ not investigated before. The binding directions in protonated polyamino receptors necessary for efficient complexation of the nitrate anion(s) were briefly outlined, as well. All these results were discussed in terms of ‘abnormal’ ^13^C chemical shift changes found previously for the side-chain carbons of amine **1** in strongly acidic solution (HNO_3_). In conclusion, an earlier proposal of its association with NO_3_^−^ at pH <1 was rejected. Instead, the participation of small amounts of a micro-species *iso*-H_4_ **1** ^4+^ **D**_hydr_ under such conditions can be proposed.

**Graphical Abstract:**

A small contribution of *iso*-H_4_ **1** ^4+^ **D**_hydr_ (see figure) to an ionic mixture of pentamine **1** was proposed to explain the ‘abnormal’ ^13^C NMR shifts observed for atoms C11 and C12 in its side-chain arm, at pH <1.
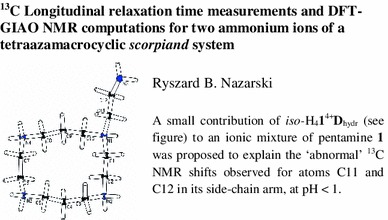

**Electronic supplementary material:**

The online version of this article (doi:10.1007/s10847-013-0298-x) contains supplementary material, which is available to authorized users.

## Introduction

While NMR chemical shifts δ_X_s (where X = C, H, etc.) and coupling constants *J*_AB_ belong to the most powerful tools available for resolution of various structural issues about organic systems, an increasing interest in the ^13^C spin–lattice (longitudinal) relaxation time *T*_1_ (hereafter referred to as ^13^C SLR and ^13^C *T*_1_) is continually observed. Because such relaxation data vary from milliseconds in macromolecules to several minutes in small objects, the ^13^C-*T*_1_ value has become an additional spectral parameter of importance to the chemist. Indeed, together with nuclear Overhauser effects arising from ^1^H decoupling of ^13^C NMR spectra, the *T*_1_ values of ^13^C nuclei permit to draw valuable conclusions about SLR mechanisms operative for individual carbon atoms in different (bio)organic systems [1–5]. It follows that they reflect both the inter- and intramolecular mobility of these entities, and so excellently complement the results on their dynamics coming from other NMR techniques such as, e.g., variable-temperature experiments. Hence, ^13^C *T*_1_s provide a reliable help in the case of some structural problems very difficult (if at all) to solve by use of more conventional methods of an NMR spectroscopy.

In our last study on macrocyclic ligands [6],^1^ the overall composite conformations of some protonated forms of a polyamine **1**, i.e., 1-(2-aminoethyl)-1,4,8,11-tetraazacyclotetradecane commonly called *scorpiand*, were proposed on the basis of its earlier ^13^C NMR pH-titration with nitric acid [7]. These spectroscopic data were analyzed in the light of GIAO (gauge-independent atomic orbitals) [8 and refs therein, 9] based predictions of δ_C_s made for numerous ammonium ions H_*n*_ **1** ^*n*+^ coexisting in aqueous media. Among other issues, we tried to explain an origin of downfield changes in δ_C_s unexpectedly observed, for atoms C11 and C12 in the *N*-pendant-arm unit of amine **1** below pH ~3.5 [7], see Fig. S1 in the Electronic Supplementary Material; an atom numbering used here is given in the Formula. As a result, the close proximity of these carbons to adjacent cationic sites at N^1^ and N^5^ in H_5_ **1** ^5+^ was suggested as one of the possibilities leading to such ‘wrong-way’ (‘abnormally’ directed) amino-protonation ^13^C NMR shifts [6]. In fact, an arrangement of the foregoing N atoms in H_5_ **1** ^5+^ would make possible, in principle, electrostatic and/or H-bonding-type attractive interactions of these cationic centers with a single nitrate anion persisting in a close vicinity of C11/C12. This kind of N^+^–H⋯O^−^–N interactions giving rise to the formation of ion pairs with NO_3_^−^ was reported for ^+^H_3_NCH_2_CH_2_NH_3_^+^ [10]. After all, it was finally concluded that the second, ‘structural’ rationalization of the observed ^13^C trends is perhaps more reliable.

Indeed, these intriguing ^13^C NMR chemical shift changes were satisfying reproduced in the time-averaged δ_C_s found for GIAO-supported overall shapes of the three subsequently formed polyammoniums H_*n*_ **1** ^*n*+^ (*n* = 3–5) [6 and refs therein]. The composite conformations of these macrocyclic ions were found, however, in a non-standard statistical analysis of the δ_C_ sets predicted for their unique promising forms. In turn, these conformers were chosen based just on the best agreement of so-computed δ_C_s with the experimental δ_C_ values. But, according to our recent work [11], large caution must be taken in interpretations of all ^13^C NMR data-based results on the shapes of molecules being in dynamic equilibrium between more than two distinct forms easily feasible energetically. Because it was also the case of the title ions H_4_ **1** ^4+^ (with non-ionized N^1^) [7, 12] and H_5_ **1** ^5+^ existing as ensembles of several fast-interconverting forms [6, 13], the three explanations of ‘anomalous’ NMR shifts in question should be considered in details (vide infra).

Thus, it became clear that the additional findings, both experimental and theoretical, on some protonated micro-species of the title system **1** were necessary. Accordingly, two sets of ^13^C-*T*_1_ times concerning internal dynamics in its ‘boundary’ ammonium cations H_*n*_ **1** ^*n*+^ (*n* = 1, 5) were determined. In addition, the scarce literature ^13^C SLR data about pendant-armed tetraaza crowns **2**–**5** were discussed in the light of current findings on these two ionic *scorpiand* species. Moreover, several low-energy conformers of the tautomeric cation *iso*-H_4_ **1** ^4+^ not analyzed before were modeled, initially with the OPLS-AA [14–17] force field and finally at the DFT level, by applying the equilibrium solvation [18] within an IEF-PCM approach [19–23]). All these results were taken into account in a renewed discussion on the origin of ‘abnormal’ NMR trends mentioned above. To the best of our knowledge, this is the first use of such *T*_1_ data for structural analysis of the protonated states of tetraazamacrocycles. Only the ^13^C-*T*_1_ based part of this work was presented in a very preliminary form [24].

## Results and discussion

### Possibilities of H-bonding between cations H_*n*_**1**^*n*+^ (*n* = 4, 5) and nitrate anion *versus* ‘wrong-way’ evolutions in NMR chemical shifts

It was obvious that host–guest interactions N^+^–H⋯O^−^–N typical of H-bond based polyammonium receptors^2^ acting as hard acids versus NO_3_^−^ as a hard Lewis base could be ruled out for the macrocyclic amine **1**, because of too small size of its intramolecular hole. Such polyaza hosts (strictly, their protonated states) showing good selectivity towards nitrate are 18- to 24-membered aza [26, 27] or oxaza crowns [28–30]. This monovalent feeble coordinating trigonal oxoanion with poor basicity offers six geometrically preferred H-bond acceptor sites according to the number and spatial arrangement of its oxygen’s lone-pair orbitals; slightly unfavorable H-bonds with the softer π-electrons are also possible [31, 32 and refs therein, 33, 34 and refs therein]. In fact, there is an extensive hydration shell around NO_3_^−^ in water [35 and refs therein] as a hard H-bonding Lewis acid [36]. Hence only specially designed macrocyclic ionophores encapsulate this anion in the aforementioned directions, by using the N–H groups in their binding pockets as strong H-bond donors [37–39]. A *C*_3_-symmetric environment in the host was found especially favorable for the NO_3_^−^ binding [37, 38, 40 and refs therein], but this intracavity orientation is not achieved for the majority of such hosts, mainly due to steric hindrance. Indeed, any strong receptor-substrate interactions result from the complementary stereoelectronic arrangement of binding sites in the host and guest [31, 37]. As a result, only half of the six preferred sites in NO_3_^−^ are usually occupied and these enable the two specific H-bonding modes involving all three or only two of its oxygens [32 and refs therein]. Similar molecular-level interactions were found very recently in the crystal structure of CH_3_CH_2_NH_3_^+^NO_3_^−^ [41].

It is also true, that while protonated polyaza macrocycles with large internal cavities can enfolded [28, 42, 43] or even encapsulated [28, 44] nitrate(s), most of the single-crystal X-ray results on such systems showed layered structures with NO_3_^−^ hovering above and below the mean planes of relatively flat receptors [26–28, 40, 42, 43]. Just such spatial arrangement was only considered for H_4_ **1** ^4+^ most likely existing in the pH range 1-4 [6 and refs therein], which would make potentially possible H-bonds with the NO_3_^−^ oxygens. The fourth protonation of **1** occurring at N^3^ [7, 12] give rise to the formation of an ‘extended’ all-out conformation of its macrocyclic unit, which most likely adopts a virtually planar macroring system, with all exocyclic N^+^-H bonds in an out configuration defined by Park and Simmons [45]. An outside orientation of the ring NH_2_^+^ groups was found for several polyammoniums of this type [6, 46–48]; see also Figs. 2, S3, and S4. Hence, a relatively rigid H-bond donor system N^2^/N^3^/N^4^/N^5^ can be considered for H_4_ **1** ^4+^. But, only its cationic site at N^5^ would be capable to interact with one discrete NO_3_^−^ ion, due to unfavorable N^+^-H bond directions at other N^+^ sites. Instead, a dual H-bond donation was likely for two neighboring cationic centers at N^1^ and N^5^ in H_5_ **1** ^5+^. So, it was only possible to think about both these ions (especially, the latter one) as entities potentially engaged in H-bonds of the type N–H⋯O^−^–N leading to the formation of supermolecules [H_4_ **1**][NO_3_]^3+^ and, particularly, [H_5_ **1**][NO_3_]^4+^ as weak 1:1 nitrate associates (ion pairs).

On the other hand, one could discuss about two other events affecting the protonated states of amine **1**, namely, (i) supporting H-bonding of type C–H⋯O^−^–N found in some crystal structures [49 and refs therein] as an equivalent of interactions C–H⋯O^−^–X (where X = C or P) known from NMR pH-titrations of some biomolecules in aqueous media [50, 51 and refs therein]. Its presence causes ‘wrong-way’ changes in the δ_H_ and δ_P_ data upon protonation to a higher and lower magnetic field, respectively. This phenomenon is perhaps electrostatic in origin and operates through the field. It was recognized as occurring internally, when a highly negatively charged group approaches the CH hydrogen(s) [51].

**Fig. 1:**
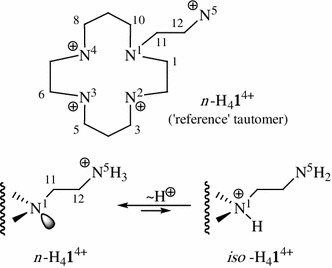
An intramolecular rearrangement possible for the tetraprotonated form of amine **1**

Moreover, there is the possibility of partial transfer of an electronic charge from N^5^ to N^1^ of a normal ion n-H_4_ **1** ^4+^ with the formation of its isomeric species iso-H_4_ **1** ^4+^ (Fig. 1) as a third (ii) explanation of ‘abnormal’ ^13^C NMR trends in question. Similar ‘wrong-way’ evolution in chemical shifts is also seen in ^15^N NMR pH-titration of unsymmetrical linear pentamines, and is explained just by equilibrium in the protonation of more than one N atom (charge delocalization) [52].

### The ^13^C relaxation times *T*_1_ for cations H**1**^+^ and H_5_**1**^5+^

In order to receive a more certain answer to the question about an origin of the ‘wrong-way’ NMR shifts mentioned above, two series of SLR times *T*_1_ of ^13^C-nuclei in the 14-membered macroring polyamine system **1** were evaluated for its aqueous solution at two pH values (11.5 and 0.2). A dedicated proton-decoupled ^13^C NMR-*T*_1_ approach and exponential data analysis were used; see Methods. The *T*_1_s determined in this way for all well-resolved ^13^C lines originating from 12 or 10 nonequivalent C sites in the mono- and pentaprotonated form of **1**, i.e., ions H**1** ^+^ (with a protonated atom N^2^) [7, 12] and H_5_ **1** ^5+^, respectively, are listed in Table 1. These relaxation data afforded valuable information on the molecular mobility of both these species, which were assumed as two ammonium cations mainly existing under such conditions. However, some contribution of the entity H_4_ **1** ^4+^ not fully protonated even at pH 0.2 can be inferred from the nonzero slope of ^13^C NMR pH-titration profiles of **1** in the pH range 0.2–1.0 [6, 7], strongly suggesting not complete protonation. In fact, an exhaustive protonation can be difficult to achievement in some cases due to an unfavorable build-up of positive charges in the macrocycle, especially if its cavity is small [53]. Such an incomplete ionization was found for several aza- and oxaza crowns [54–56].

**Table 1:** Experimental ^13^C longitudinal relaxation times, *T*_1_s, determined for the atoms C1–C12 in the ions H**1** ^+^ and H_5_ **1** ^5+^, s ^a^

Carbon no.^b^	H**2** ^+^ [pH 11.5]	H_5_ **2** ^5+^ [pH 0.2]
1	0.46 (2)	0.29 (2)
2	0.42 (1_5_)	0.27 (2)
3	0.45 (2)	nd^c^
4	0.42 (2)	0.38 (4)
5	0.49 (3)	0.29_5_ (1_5_)
6	0.40 (1)	0.32 (3)
7	0.41 (2)	0.30 (2)
8	0.41 (1)	nd^c^
9	0.39 (2)	0.36 (4)
10	0.47 (2)	0.32 (2)
11	0.49 (4)	0.38_5_ (4_0_)
12	0.65 (3)	0.67 (6)

^a^Values given in the parenthesis are ± errors in the last significant figure

^b^For atom numbering see Fig. 1

^c^Not determined due to practical overlapping the ^13^C NMR lines coming from C3 and C8

In any NMR experiment, irradiated nuclei transfer their excess spin energy to the surrounding in a process of spin–lattice relaxation (SLR), which rate *R*_1_ can be expressed by the sum of pertinent reciprocal relaxation times *T*_1_^−1^ employing Eq. 1 [1–5].

1 $$ R_{ 1} = { 1}/T_{ 1} = { 1}/T_{{ 1,{\text{DD}}}} + { 1}/T_{{ 1,{\text{other}}}} $$

Of the four mechanisms of ^13^C SLR possible for isotropic solutions of diamagnetic systems with typical (spin *I* = ½) NMR nuclei [dipolar (dipole–dipole, DD), spin-rotation, chemical-shift anisotropy, and scalar coupling], we can expect that an intramolecular DD relaxation provides the dominant effect for pentamine **1** because each of its C atoms carries two attached protons. Indeed, an overwhelming predominance of this mechanism for CH_2_ carbons in structurally close macrocyclic tetramines **2**–**5** [57, 58] and polyethers [59] was previously found, by measuring the ^13^C-{^1^H} Overhauser enhancement factors (*η*_obsd_ + 1) and calculating the purely DD contributions to relevant ^13^C *T*_1_s, by using Eq. (2) (where *η*_max_ = γ_H_/2γ_C_ = 1.988).

2 $$ T_{{ 1,{\text{DD}}}} = T_{ 1} (\eta_{ \hbox{max} } /\eta_{\text{obsd}} ) $$

For such molecules rapidly reorienting isotropically in a liquid phase (solution or neat) under ^1^H decoupling and ‘extreme narrowing limit’ conditions, the DD relaxation rate of the ^13^C nucleus *i* is very well approximated by Eq. 3

3 $$ \left( { 1/T_{{ 1,{\text{DD}}}} } \right)_{i} \,=       N\hbar^{ 2} \gamma_{\text{C}}^{ 2} \gamma_{\text{H}}^{ 2} r_{\text{ij}}^{ - 6} \tau_{{{\text{c}},{\text{eff}}}} = {\text{ constant}} \times N\tau_{{{\text{c}},{\text{eff}}}} $$

in which $\hbar$ is reduced Planck’s constant (≡*h*/2π), γ’s are the gyromagnetic ratios of ^13^C and ^1^H, *τ*_c_ is the molecular correlation time, *r*_ij_ is an effective C_*i*_-H_*j*_ internuclear distance (~1.09 Å), and *N* is the number of adjacent protons *j*, because contributions to ^13^C SLR from the other protons are practically negligible, due to the *r* ^−6^ dependence [1–3, 5, 60]. But, such an overall tumbling cannot easily by resolved into its components (translation, vibration, rotation) and the average time taken between two reorientations is defined as an effective correlation time *τ*_c_. The proportionality 1/*T*_1,DD_ ∝ *τ*_c_ is expressed in a rule *the faster a molecule, the longer is* *T*_1_ (and shorter *τ*_c_), as all carbons within a given system move at the same rate. Most of the nonviscous small and medium-sized rigid objects fulfils this condition. However, conformationally flexible systems are usually anisotropic in their tumbling and related *τ*_c,eff_s can be different for each of their C atoms. The *NT*_1_ value is then no longer a constant, but inversely proportional to *τ*_c,eff_ (Eq. 4)

4 $$ \left( {NT_{ 1} } \right)_{i} \, \propto \left( { 1/\tau_{{{\text{c}},{\text{eff}}}} } \right)_{i} $$

and this quantity can be interpreted as an internal mobility parameter, although only qualitatively and with caution [5]. Indeed, besides an overall tumbling, the flexible molecules (such the system **1**) may have many modes of internal mobility, e.g., segmental dynamics along a side arm or conformational macroring inversions. Each of these motions modulate the DD interaction between coupled nuclei.

From the foregoing, it follows that the calculated *τ*_c,eff_ or *NT*_1_ data are generally considered as reliable measures of both the *mobility* [of the whole molecule (overall tumbling) and/or its sub-units (segmental mobility)] and the *ordering* [1–5, 60]. In our case, all numerical ^13^C *T*_1_ values found for two ions H_*n*_ **1** ^*n*+^ can be directly compared, because only CH_2_ groups exist in these species (*N* = 2). Hence, the gross consideration of measured SLR data was applied as completely sufficient for the purpose of our analysis. Moreover, their overall description exclusively in terms of a DD mechanism appears appropriate. The same approach was used in the work [57].

In contrast to the ^13^C-*T*_1_ results of Wyrwał et al. [58] on non-protonated systems of *cyclam* (**3**) and its two derivatives **4** and **5**, where all macroring backbone carbons can be treated as dynamically equivalent in CDCl_3_ solution, analogous atoms in both unsymmetrical ions H_*n*_ **1** ^*n*+^ (*n* = 1 or 5) studied here in water are rather diverse in this respect, especially in strongly acidic medium. Generally, the magnitudes of related ^13^C-*T*_1_ values found for these two ions are between those reported for azacrowns **3** and **4** [58], whereas the shortening of such data for H_5_ **1** ^5+^relative to H**1** ^+^ indicates a slower overall tumbling of the former one.

As one can easily see, the mobility of CH_2_ groups in pendant-arms ^α^CH_2_^β^CH_2_NH_2_ and ^α^CH_2_^β^CH_2_NH_3_^+^ of these ions increases with an increasing distance from the macrocycle center (*T*_1_s becomes longer, Table 1). In both cases, *T*_1_s estimated for α-Cs are equal to the greatest value found for ring carbons, whereas these parameters for β-Cs are identical within the error limits (~0.66 s) and, simultaneously, they are the longest ones among all of these relaxation rates. Side-chain segmental motion was apparent by the lengthening *T*_1_s along both aminoalkyl groups toward their ^β^CH_2_N terminus. A pronounced degree of such motion, typical for open-chains, was also reported for the side arms of **2** and **4** [57, 58]. Indeed, the mean *T*_1_ values for macroring carbons in two ions H_*n*_ **1** ^*n*+^ (of 0.43 and 0.32 s for *n* = 1 and 5, respectively) can be expected to approximate the overall *T*_1_s of these species. It was obvious that greater ^1^ *T*_1_s found for all four *N*-pendant-armed systems mentioned above are due to an added internal motion, i.e., an enhanced segmental freedom of their side chains.

The *T*_1_ value of 0.67 ± 0.06 s, i.e., 2.0 × ~0.32 s (estimated for the ring), found for C12 in H_5_ **1** ^5+^ is greater than ~0.48 s predicted from simple comparison with related data for the more mobile H**1** ^+^ (vide supra). However, this *T*_1_(β-C)/*T*_1_(ring) ≅ 2.0 is fully consistent with the analogous *T*_1_/*T*_1_ ratio of 2.25 found in D_2_O solution for the ^α^CH_2_^β^CH_2_OH unit of **2** [57]. Moreover, our results indicate much faster internal rotation of the β-CH_2_ group in **1** at pH 0.2, in agreement with an enhanced mobility awaited for this site in the ^α^CH_2_^β^CH_2_NH_3_^+^ unit solvated by ion–dipole interactions in strongly polar aqueous solution [60].

The conformational flexibility of an internal hole of H**1** ^+^ evaluated in this manner is in good agreement with the average experimental vicinal interproton coupling ^3^ *J*_HH_ of ~5.3 Hz. This *J*-value, typical of rapidly interconverting cyclic systems, was estimated in a first-order analysis of ring proton multiplets appeared in the 500 MHz ^1^H NMR spectrum of **1** recorded at pH 11.5 [12]. On the other hand, intramolecular H-bonds to adjacent ring nitrogens (or even being in a dynamic H^+^-exchange between two such atoms, NH⋯H^+^⋯HN) [61] are highly probable at this protonation state. Consequently, an internal fluctuation of CH_2_ protons in the macrocyclic backbone of H**1** ^+^ is always slower than the mobility of such protons in its side-chain. Similar situation, reflected by comparable magnitudes of ^13^C *T*_1_s or substantial line broadening of ^1^H NMR signals, was also reported for other *N*-pendant-armed azacrowns [58, 62].

In turn, relative small mobility of **1** in its strongly acidic solution is in line with similar observations made for other polyhetero macrocycles, which usually are preorganized structures with specific segmental conformations. To bind metal cations or protons they may change the shape of each ring segment, thereby reducing the *T*_1_s [59 and refs therein]. A low mobility of H_5_ **1** ^5+^ most likely results from strong distance-dependent Coulombic-type repulsions between four positively charged ammonium sites at N^1^–N^4^ as electrostatic solute ordering effects, which ‘fix’ its macrocyclic core in a maximally ‘extended’ form adopting an all-out conformation with ring *N*-atoms occupying four corners of the molecular polygon and N^+^-H bonds directed toward the outward of an internal cavity (vide supra). An additional ‘ordering’ can results from interactions between ring cationic sites and their counter ions or solvent shell of an aqueous surrounding. All such phenomena have a strong effect on the *τ*_c_ value [63].

The aminoalkyl side chain of the monoprotonated base, H**1** ^+^, was recognized previously as its highly mobile fragment. Indeed, the ‘medium’ coupling ^3^ *J*_HH_ ~7.1 Hz, a signature for the fast conformational interconversion [64], was estimated at pH 11.5 [12]. In other words, there is a typical ‘freely’ rotating ethane unit [65]. A practical equivalence of *T*_1_s found for terminal atoms C12 in pendant arms of two discussed ions of **1** indicates that the analogously fast rotation also occurs around the single bond CH_2_-CH_2_NH_3_^+^ in H_5_ **1** ^5+^. Obviously, similar mobility of β-CH_2_ groups in both these species suggests similar solute–solvent interactions of their outer side chains with an aqueous environment. For important implications of this conclusion, see below.

As has already been mentioned, the ‘wrong-way’ ^13^C NMR pH-titration shifts found for **1** at pH <3.5 were reproduced quite well by δ_C_s predicted for effective overall (population-weighted averaged) shapes of the main forms of cations H_*n*_ **1** ^*n*+^ (*n* = 3–5) coexisting in an acidic medium [6]. The proposed multicomponent conformations of these composite shapes called H_3_ **1** ^3+^ **ABCD**, *n*-H_4_ **1** ^4+^ **BC** and H_5_ **1** ^5+^ **ABCD** were, in turn, elucidated by the best fitting measured δ_C_s to pertinent theoretical δ_C_ data computed by the GIAO B3LYP/6-31G(*d*) method. Strictly, the NMR shift of a given C atom, for all of these overall structures, was obtained as a weighted average δ_C_ value of the same atom in a few preselected forms sampled by a conformational search at the DFT level. For that reason, the whole analysis was a little arbitrary, but it was only one approach possible at this research stage. Nonetheless, in view of the present ^13^C-*T*_1_ results on internal dynamics in H**1** ^+^ and H_5_ **1** ^5+^, one can accept that an intermolecular H-bond of type N^+^–H⋯O^−^–N (hypothetically considered before [6], in particular for H_5_ **1** ^5+^) does not operate in aqueous solution. Without any doubt, such nitrate complexation, giving rise to the formation of a supermolecule [H_5_ **1**][NO_3_]^4+^, would substantially enforce the rigidity of the pendant-arm unit in H_**5**_ **1** ^5+^. However, the anticipated [57, 66 and refs therein] slowdown of internal dynamics of its two constituent CH_2_ groups rooted by H-bonding mentioned above, was not found.

The above conclusion is consistent with other considerations. Indeed, a close inspection of low-energy forms of H_4_ **1** ^4+^ and, especially, H_5_ **1** ^5+^, which were recognized as contributing to their composite shapes H_4_ **1** ^4+^ **BC** and H_5_ **1** ^5+^ **ABCD** [6], indicates that ammonium sites in these protonation states of **1** do not fulfill the highly specific spatial requirements of the interactions N^+^–H⋯O^−^–N necessary for efficient complexation of nitrate ion (vide supra). Moreover, the supporting H-bonds C–H⋯O^−^–N are not possible.

### Prediction of NMR spectra for the tautomer *iso*-H_4_**1**^4+^

Amines characteristically exhibit small upfield or even weak downfield protonation shifts for the C atoms α to N atoms and mostly large high field shifts for β-carbons, in ^13^C NMR spectra [12, 67 and refs therein]. During the protonation of N^1^ as a weakest basis center in pentamine **1**, two β-carbons in the ring, i.e., C2 and C9, show typical upfield changes at pH <1.5 while side-chain atoms C12 and especially C11 behave abnormally [7] (Fig. S1). According to all foregoing facts, a prototropic rearrangement shown in Fig. 1 would excellently rationalize these ‘abnormal’ trends observed. Indeed, deprotonation of some N atoms, at the expense of protonation of others in close enough proximity and accompanied by differently directed ^13^C NMR shifts, was reported for both open-chain [68, 69] and macrocyclic [67, 70–72] polyamines. Such type ‘wrong-way’ protonation effects in the multinuclear NMR pH-titrations were sporadically reported for a great variety of small to large molecules possessing basic sites [73 and refs therein].

Thus, several conformers of a tautomeric ion *iso*-H_4_ **1** ^4+^ not studied to date, with the protonated N^1^–N^4^, were generated applying the OPLS-AA [14–17] force field successfully used previously for normal ions *n*-H_*n*_ **1** ^*n*+^ [6]. The resulting models of *iso*-H_4_ **1** ^4+^ (Table S1) were refined in further quantum–mechanical DFT-level calculations, involving an IEF-PCM hydration simulation, evaluation of DFT-D3 [74] corrected energies, and GIAO-based predictions of NMR spectra (Methods). Because standard density functionals do not describe correctly the intramolecular electron-correlation interactions attributed to van der Waals dispersion forces [74, 75],^3^ the adequate DFT-D3 corrections to DFT energies (more precisely, related *Δ* *G*_298.15_^o^ data) were also evaluated for final B3LYP/6-31(*d*)-optimized structures; similar approach was used in two recent papers [11, 76]. All important results found in this way for the low-energy forms **A**–**D** of *iso*-H_4_ **1** ^4+^ are given in Table S3.

**Fig. 2:**
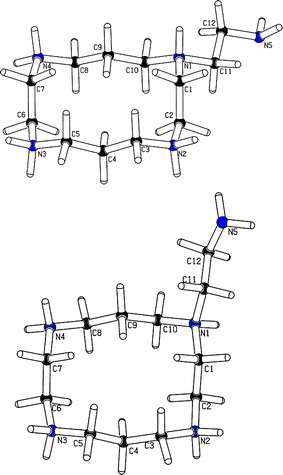
PLATON views of two B3LYP/6-31G(*d*)-optimized lowest energy ‘hydrated’ forms **A** (*top*) and **D** (*bottom*) of *iso*-H_4_ **1** ^4+^; all N atoms are shown in* blue*

The aforementioned conformers of *iso*-H_4_ **1** ^4+^ were recognized as species of higher energy than related forms of *n*-H_4_ **1** ^4+^ attained in predominant protonation of atoms N^2^–N^5^. These forms of *iso*-H_4_ **1** ^4+^ with an all-out topology of N–H bonds attached to ring nitrogens were found similar to those established for H_5_ **1** ^5+^ [46–48]. But, strongly elongated bond C11–N^1^ of ~1.585 Å, shorted bond C12–N^5^ (~1.442_5_ Å), and slightly flattened amino site at N^5^ were unexpectedly found for its lowest-energy form **A** with the outer unit –CH_2_CH_2_N^5^H_2_ in an equatorial position (Fig. 2). Analogous geometry of the axially oriented side-chain R was found also for *iso*-H_4_ **1** ^4^ **B** (*Δ* *E*_tot_ = 2.47 kJ mol^−1^, *Δ* *G*_298.15_^o^ = 1.81 kJ mol^−1^) and two forms **C** and **D** with R_eq_ and R_ax_, respectively (Table S3). An increase in pyramidality at N^5^ on going from **A** to **D**, expressed by the sum of valence angles around this nitrogen,^4^ was also remarkable. However, all these results on *iso*-H_4_ **1** ^4+^ were predicted for a physically unreal case of isolated polyammoniums in the gas phase at 0 K, while experimental data were determined for their strongly polar aqueous solutions at ~294 K. Indeed, various effects of crucial importance such as interactions with counterions, solvation, thermal effects, etc. were completely ignored in this standard approximation ‘of the free-molecule’.

Consequently, simulations of an impact of water molecules on the shape of *iso*-H_4_ **1** ^4+^ were undertaken, by using an improved IEF-PCM protocol (Methods). As expected, its abnormal gas-phase geometry strongly changed after such ‘dissolution’ in water. The resulted forms **A**_**hydr**_ **-D**_**hydr**_, of *iso*-H_4_ **1** ^4+^ possess all above bonds of normal length (Table S3). A large relaxation of their geometry around the C11–N^1^ bond is noteworthy, in particular.

Obviously, much more important were NMR properties of such constructed conformers of *iso*-H_4_ **1** ^4+^. Thus, four pairs (*iso*-H_4_ **1** ^4+^, *n*-H_4_ **1** ^4+^) of the structurally close ions were considered in order to compute differences in δ_C_s arising from the change *n*-H_4_ **1** ^4+^ → *iso*-H_4_ **1** ^4+^. All forms **A**–**D** of *iso*-H_4_ **1** ^4+^ were found as transforming themselves into related conformers **N1** and **N2** of *n*-H_4_ **1** ^4+^ (for their ‘hydrated’ states, see Figs. S3 and S4) used as ‘reference’ systems with the same ring geometry, i.e., with protonated N^1^ and unprotonated N^5^ (Table S2). As a result, two narrow intervals *Δ*δ_C_^calc^ of +(13.9–15.5) and −(2.2–3.2) ppm were in vacuo GIAO-predicted for the atoms C11 and C12, respectively. This trend was in qualitative agreement with an alteration of +0.90 and −0.34 ppm found experimentally for the pH change from 1.02 to 0.24 [6 and refs therein]. But, analogous B3LYP/6-31G(*d*) IEF-PCM (H_2_O) NMR predictions on ‘hydrated’ forms **A**–**D**, *Δ*δ_C_^calc^ of +(6.6–9.8) and −(1.9–3.2) ppm, were in much better conformity with the experiment, particularly in the magnitude of the trend (Table S3). An impact of the geometry relaxation around C11 is evident. In particular, this concerns the thermodynamically preferred forms **A**_**hydr**_ and, especially, **D**_**hydr**_ with the DFT-D3 corrected *Δ* *G*_298.15_° of 1.4 and 0.0 kJ mol^−1^, respectively.

Interestingly, both conformers **N1** and **N2** of H_4_ **1** ^4+^ were previously recognized as forms most favored in the gas-phase [6], but they were not proposed finally as existing in the real aqueous medium on the basis of a ‘*solution environment* (i.e., NMR spectroscopic) *match criterion*’ [6, 11, 78]. Indeed, several forms of some multi-component systems initially located as their global energy minima were occasionally not recognized in solutions, by using typical GIAO-supported approaches [6, 78–80]. The majority of discrepancies of this kind was usually explained by specific solute–solvent effects only seldom adequately taken into account in the computational treatment in normal use. Our recent results on multi-conformer mixtures [11] and the present findings on H_*n*_ **1** ^*n*+^ permit to be skeptical a bit about the quantitative reliability of current standard experimental versus computational NMR-data-based protocols for some flexible systems, especially those for which only δ_C_s are used in their conformational analysis. For instance, a presumable uncertainty of such labor-consuming evaluations of the compositions of equilibrium mixtures of different forms of H_*n*_ **1** ^*n*+^ in aqueous solution was of the order of 10–15 % [6].

In view of the foregoing, one can consider the presence of small amounts of the **D**_**hydr**_ and **A**_**hydr**_ forms of *iso*-H_4_ **1** ^4+^ equilibrated with the **N1**_**hydr**_ and **N2**_**hydr**_ forms of *n*-H_4_ **1** ^4+^, respectively, in the ionic mixture of **1** at pH <1. Indeed, the full protonation of this pentamine was only arbitrarily assumed previously (vide supra, see also note 71 in Ref. [6]). On the other hand, a ‘structural’ rationalization [6] of the discussed ^13^C trends agreed well with a reasonable postulate that both atoms C11 and C12 have been in a comparable chemical environment under used measurement conditions. The latter assumption resulted, in turn, from large similarity in the shape of their NMR pH-titration profiles (*resemblance criterion*) [12]. A presumable coexistence of some minor amounts of *iso*-H_4_ **1** ^4+^ being in dynamic equilibrium with *n*-H_4_ **1** ^4+^ is consistent with such conformational landscape.

Generally, the higher energies of localized forms of *iso*-H_4_ **1** ^4+^ in relation to those of *n*-H_4_ **1** ^4+^ seem to be the only one alarming aspect of a newly proposed explanation of ^13^C NMR shifts in question. However, it must be kept in mind that we meet here with the well-known issue of a doubtful trustworthiness of today’s computational predictions about multicharged polyammoniums dissolved in highly polar aqueous media. Moreover, the presence of NO_3_^−^ as counterions was neglected. Similar relaxation times *T*_1_ (~0.66 s) estimated for C12 of **1** at pH 11.5 and 0.2 suggests similarity in their dynamics and so comparable solute–solvent interactions of its pendant arm in two different surroundings. The occurrence of the same molecular unit ^α^CH_2_^β^CH_2_NH_2_ in H**1** ^+^ and *iso*-H_4_ **1** ^4+^ would ideally explain practically identical ^13^C-*T*_1_ values found for their β-CH_2_ groups.

## Conclusion

A crucial role of the ^13^C spin–lattice relaxation times (^13^C *T*_1_s) for elucidating internal molecular dynamics was presented in the case of two ammonium cations of a complex tetraazamacrocyclic *scorpiand* (**1**) system studied by this NMR technique in aqueous medium. These experimental *T*_1_ data, in conjunction with the DFT-level GIAO-based prediction of ^13^C NMR chemical shifts carried out for several conformers of the ion *iso*-H_4_ **1** ^4+^ not studied before, permitted to suggest the presence of minor amounts of this tautomer in solution, as a species co-existing in fast equilibrium with *n*-H_4_ **1** ^4+^. Such contribution of *iso*-H_4_ **1** ^4+^ to the ionic mixture would rationalize, at least in part, an ‘abnormal’ ^13^C NMR trend found previously for the side-chain atoms C11/C12 in **1** below pH 1. At the same time, its earlier working explanation, involving complexation of a single nitrate anion by the perprotonated form of pentamine **1** was rejected in a definitive manner.

## Methods

### ^13^C NMR relaxation measurements

Longitudinal relaxation times, *T*_1_s, for ^13^C nuclei in amine **1** (available from an earlier work [7]) were measured at ~294 K on undegassed samples by the inversion-recovery method [81, 82] on a Varian Gemini 200 BB NMR spectrometer operating at 199.98/50.29 MHz (^1^H/^13^C). All experiments were conducted in automation mode under ^1^H broad band-decoupling conditions achieved with the WALTZ-16 sequence [83], by using pulse program of the software package (version 6.3C) from Varian Associates, Inc. The (*t*_d_-π-*τ*-π/2-*t*_a_)_*n*_ pulse sequence was applied, where *t*_d_, *τ*, and *t*_a_ were the recycle-delay time, relaxation delay, and acquisition time, respectively. Twelve different pulse interval times *τ* between 0.01 and 20 s were used in arrayed experiments, with *t*_d_ 20 s and *t*_a_ 4.2 s. Number of scans, *n*, was between 400 and 900, spectral width 3200 Hz, data size 32 K.

High-precision 5-mm NMR sample tubes were used. The δ_C_ values, originally measured relative to external liquid tetramethylsilane (TMS) [contained in a coaxially-situated glass NI5CCI-V insert (with the 2-mm-o.d. stem) delivered by Norell, Inc. Landisville, NJ, USA], were corrected by a factor of +0.72 ppm [12], to account for the difference in diamagnetic susceptibilities of both liquids involved (*Δ*χ_v_) [84 and refs therein]. Roughly 0.01 mol L^−1^ solution of **1** in H_2_O/D_2_O (~95:5 vol. %) was applied and HNO_3_ was employed as titrant; the concentration of **1** and D_2_O decreased a little, because of dilution of the sample with the added acid. Two solutions of pH values about 11.5 and 0.2 were studied; pH-meter readings were not corrected for a small isotope effect of D_2_O presents [85]. For details of pH-metric measurements, see Ref [7]. The *T*_1_s for ^13^C nuclei in the ions H**1** ^+^ and H_**5**_ **1** ^5+^ were estimated with the aid of two-parameter non-linear least-squares fitting program provided by the Varian NMR system. All calculations were carried out on a spectrometer processor.

### Molecular modeling and prediction of NMR spectra

An exhaustive molecular-mechanics (MM) exploration of the conformational space of *iso*-H_4_ **1** ^4+^ was performed with the OPLS-AA [14–17] force field as an energy minimizer, by using the Monte Carlo (MC)-type GMMX subroutine of PCMODEL [86]. A randomization [87, 88 and refs therein, 89] over various macroring conformers and all rotatable bonds in the side chain was performed. The 14.6 kJ mol^−1^ energy window and dielectric constant (bulk relative permittivity), ε = 78.36 [90], were used in a rough simulation of hydration.^5^ The returned 25 unique energetically lowest-lying models of *iso*-H_4_ **1** ^4+^, embracing the energy window of 6.2 kJ mol^−1^, were subjected to a gradient gas-phase geometry refinement, initially at HF/3-21G [91] and then (after some selection) at HF/6-31G(*d*) and B3LYP/6-31G(*d*) levels, by applying the Gaussian 09 program [90] with PCMODEL as its graphical interface. Seven HF/3-21G promising trial structures **A-F** of *iso*-H_4_ **1** ^4+^ found in this way are listed in Table S1.^6^ In contrast, all input MM models of the likewise examined ‘reference’ forms **N1** and **N2** of *n*-H_4_ **1** ^4+^ were attained departing from geometries of two structurally close forms of *iso*-H_4_ **1** ^4+^, by their manual deprotonation at N^1^.

In addition, frequencies *ν*_i_ were always computed in harmonic approximation of vibrational modes to verify whether all localized stationary points represented true energy minima (NImag = 0) and to determine differences in standard Gibbs free energies at 298.15 K, *ΔG* ^o^_298.15_. Zero-point energies were evaluated from *ν*_i_s scaled by a uniform factor of 0.96 [93]. Finally, Grimme’s DFT-D3 corrections [74] for dispersion-type interactions (London forces) [74, 75] were applied to so-computed *ΔG* ^o^_298.15_s. These correcting terms were calculated with ORCA [94]. Moreover, simulations of an impact of water molecules on the shape of ions **1** were performed in an improved equilibrium solvation protocol [18] of the polarizable continuum model of solvation (IEF-PCM) [19–23], by using UFF atomic radii. All molecule visualizations were performed employing PLATON [95–97].

Single-point in vacuo GIAO [8, 9] B3LYP/6-31G(*d*) computations of isotropic magnetic shieldings, σ_C_s, for components of all four ionic pairs of **1** were carried out at their B3LYP/6-31G(*d*) ground-state structures, by using Gaussian 09. Analogous predictions were also made applying the foregoing hydration model. The ^13^C NMR chemical-shift value of a given nucleus in all these entities was defined as δ_C_^calcd^ [ppm] = σ_C_^stand^ − σ_C_^calcd^, where σ_C_^stand^ was of 189.7155 ppm (in vacuo) or 190.1647 ppm (IEF-PCM simulations of H_2_O) as respectively evaluated for a used NMR reference standard (TMS with the *T*_d_ symmetry) [98]. All final geometry optimizations, frequency calculations, and GIAO predictions at the DFT level were done with the keyword Int(Grid = UltraFine).

## Electronic supplementary material

Below is the link to the electronic supplementary material.

Transformations of 25 initial MM models of *iso*-H_4_ **1** ^4+^ in HF/3-21G calculations, the key energetic, structural, and GIAO-based NMR data for the forms **A-D** of *iso*-H_4_ **1** ^4+^ and **N1** and **N2** of *n*-H_4_ **1** ^4+^ as ‘reference’ systems (including the IEF-PCM/H_2_O results), energetic data, and Cartesian coordinates for all conformers under study. Correction to Ref. [6] is also added. Figs. S1-S5, Tables S1-S15 (19 pages). (PDF 345 kb)

## Footnotes

1 A certain erroneous explanation given in Ref. [6] about atoms C3–C7 and the N^3^ site in two cations H_*n*_ **1** ^*n*+^ was corrected in the Electronic Supplementary Material (p. S7).

2 For an excellent review in the field of polyammoniums, see e.g., Ref. [25].

3 For other papers concerning this topic, see e.g., note 45 in Ref. [11].

4 Such an approach was used inter alia in Ref. [77].

5 Calculations were executed for up to 210000 MC steps. A search was carried out in seven sets of the MM runs, where every series embraced 30000 MC steps; no new low-energy structure was generated in the last set.

6 Two starting MM models of *iso*-H_4_ **1** ^4+^, Nos. 22 and 23, with the bent –CH_2_CH_2_NH_2_ unit, underwent rearrangement into forms **X** and **Y** of *n*-H_4_ **2** ^4+^ via the 5-membered cyclic transition state (observed on the monitor screen) with an internal H-bridge of type N⋯H^+^⋯N. A great degeneration of initial MM models of flexible molecules occurs occasionally in the geometry refinement at different ab initio levels. Usually, some changes in relative positions of their energy levels appear in such cases, e.g., #1 → **E**, #2 → **F**, #13 → **A**, #14 → **B** (Table S1); similar observation was reported in Ref. [92].
