# Supplementary material for: 13C longitudinal relaxation time measurements and DFT-GIAO NMR computations for two ammonium ions of a tetraazamacrocyclic scorpiand system
Source: J Incl Phenom Macrocycl Chem. 2013 Mar 2;78(1):299–310. doi: 10.1007/s10847-013-0298-x (PMC3887262; doi:10.1007/s10847-013-0298-x)
Supplement: Supplementary file 1 — Transformations of 25 initial MM models of iso-H4 1 4+ in HF/3-21G calculations, the key energetic, structural, and GIAO-based NMR data for the forms A-D of iso-H4 1 4+ and N1 and N2 of n-H4 1 4+ as ‘reference’ systems (including the IEF-PCM/H2O results), energetic data, and Cartesian coordinates for all conformers under study. Correction to Ref. [6] is also added. Figs. S1-S5, Tables S1-S15 (19 pages). (PDF 345 kb) [file 10847_2013_298_MOESM1_ESM.pdf]

Electronic Supplementary Material

Physical image *vs.* molecular structure relation. Part 16 – ^{13}C Longitudinal relaxation time measurements and DFT-GIAO NMR computations for two ammonium cations of a tetraazamacrocyclic *scorpiand* system

Ryszard B. Nazarski

Laboratory of Molecular Spectroscopy, Faculty of Chemistry, University of Łódź, Tamka 12, 91-403 Łódź, Poland
E-mail: nazarski@uni.lodz.pl

Table of Contents:

Ad ^{13}C NMR spectra / Molecular modeling

Figure S1. ^{13}C NMR pH-titration profiles for amine 1 in H_2O/D_2O S2
Table S1. Transformations of 25 initial MM models of *iso*-H_41^{4+} observed in the HF/3-21G level computations S3
Figure S2. A graphical representation of transformations of 25 initial MM models of *iso*-H_41^{4+} during the HF/3-21G computations S4
Table S2. Key energetic, structural and NMR data for conformers N1 and N2 of the cation *n*-H_41^{4+} (as ‘reference’ systems) S4
Table S3. Important energetic, structural and NMR data computed for the forms A-D of *iso*-H_41^{4+}........ S5
Figure S3. PLATON views of *n*-$H_41^{4+}N1_{hydr}$ being in a postulated equilibrium with the conformer A_{hydr} of *iso*-H_41^{4+}........ S6
Figure S4. PLATON views of *n*-$H_41^{4+}N2_{hydr}$ being in a postulated equilibrium with the conformer D_{hydr} of *iso*-H_41^{4+}........ S6
Figure S5. ^{13}C{1H} NMR spectrum of amine 1 in H_2O/D_2O at pH 2.05 (Correction of an earlier explanation given in Ref. [6]) S7

Ad Final DFT-level structures

Tables S4 – S15. Cartesian coordinates and energy data for all important conformers of cations H_n1^{n+} under this study S8-S19

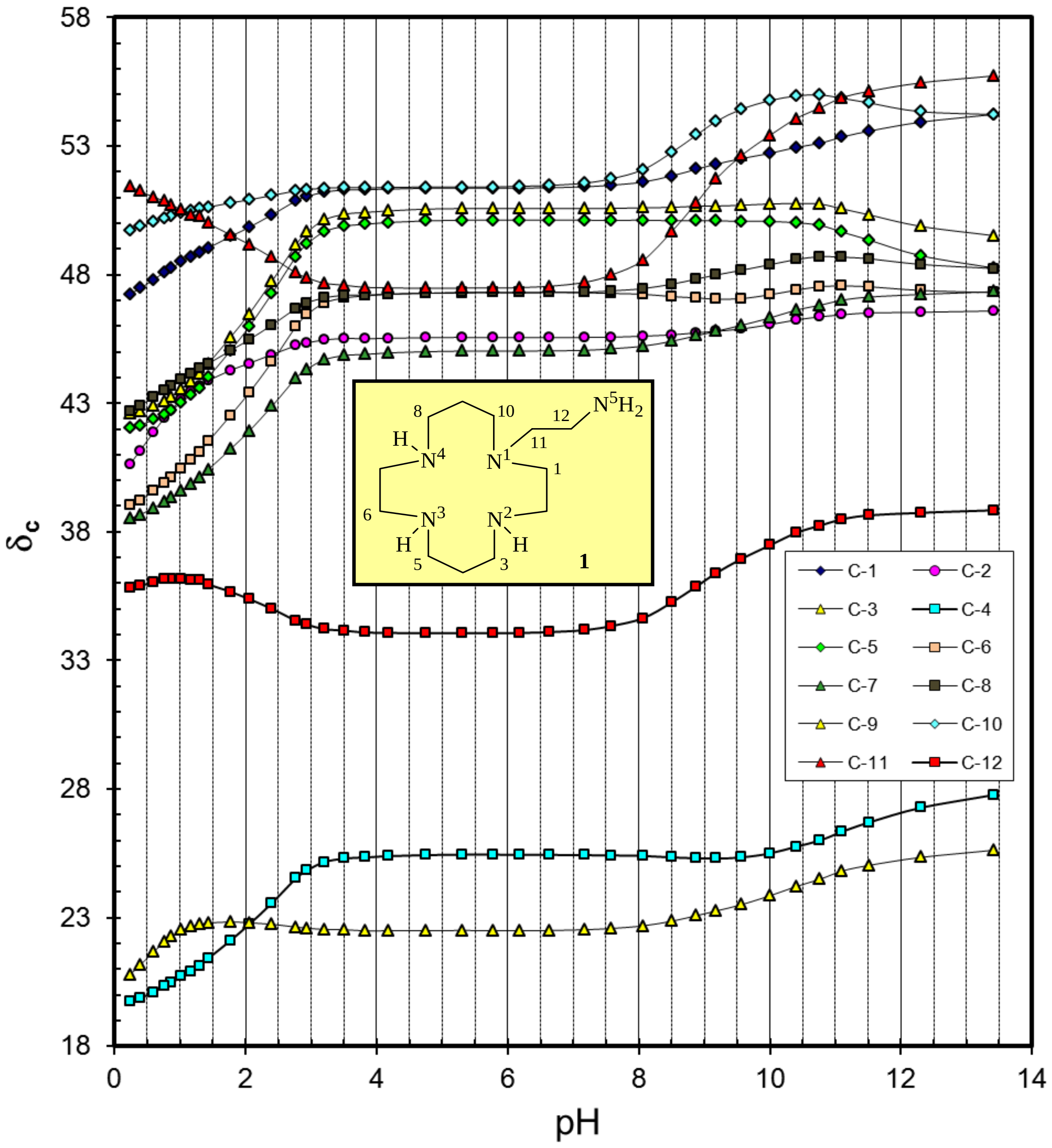

Figure S1. 50.29 MHz $^{13}C\{^1H\}$ NMR pH-titration curves for ~0.01 mol L^{-1} solution of pentamine **1** in H_2O/D_2O (~95:5 v/v) at ~21°C; isotope effect for pH neglected. For other experimental details, see Refs. [7,12] in the main text. The representative spectrum registered at pH 2.05 is shown in Fig. S5.

Table S1. Transformations of 25 initial MM models[a] of *iso*-H_4**1**$^{4+}$ during *ab initio* computations at the HF/3-21G theory level

Initial MM form No.	OPLS-AA force field energy, kcal mol^{-1}	Relative energy, kJ mol^{-1}	HF/3-21G energy, Ha mol^{-1}	Relative energy, kJ mol^{-1}	Final name of the conformer
**1**	**-13.351**	**0.00**	-740.280007	21.82	**E**
**2**	-13.221	0.54	-740.278096	26.84	**F**
**3**	-13.192	0.67	**-740.288318**	**0.00**	**A**
**4**	-13.186	0.69	-740.287276	2.74	**B**
**5**	-13.160	0.80	-740.278096	26.84	**F**
**6**	-13.013	1.41	-740.286736	4.15	**C**
**7**	-12.962	1.63	-740.280007	21.82	**E**
**8**	-12.953	1.67	-740.286072	5.90	**D**
**9**	-12.941	1.72	-740.280007	21.82	**E**
**10**	-12.822	2.21	-740.287276	2.74	**B**
**11**	-12.822	2.21	-740.288318	0.00	**A**
**12**	-12.820	2.22	-740.278096	26.84	**F**
**13**	-12.816	2.24	-740.288318	0.00	**A**
**14**	-12.807	2.28	-740.287276	2.74	**B**
**15**	-12.774	2.41	-740.278096	26.84	**F**
**16**	-12.753	2.50	-740.278096	26.84	**F**
**17**	-12.646	2.95	-740.286736	4.15	**C**
**18**	-12.626	3.03	-740.286736	4.15	**C**
**19**	-12.589	3.19	-740.286072	5.90	**D**
**20**	-12.574	3.25	-740.286072	5.90	**D**
**21**	-12.238	4.66	-740.275055	34.82	**G**
**22** [b]	-11.904	6.05	**-740.355728**	**-176.98**	**Y** [c]
**23** [b]	-11.896	6.09	**-740.357762**	**-182.32**	**X** [c]
**24**	-11.874	6.18	-740.275055	34.82	**G**
**25**	-11.868	6.20	-740.275055	34.82	**G**

[a] Optimizations were performed using the OPLS-AA force field with simple simulation of hydration, ε = 78.39 (Methods, in the main text). [b] The MM model with the bent $–CH_2CH_2NH_2$ unit, which underwent an internal rearrangement *iso*-H_4**1**$^{4+}$ → *n*-H_4**1**$^{4+}$. [c] The conformer of the resulted cation *n*-H_4**1**$^{4+}$ of considerably lower energy.

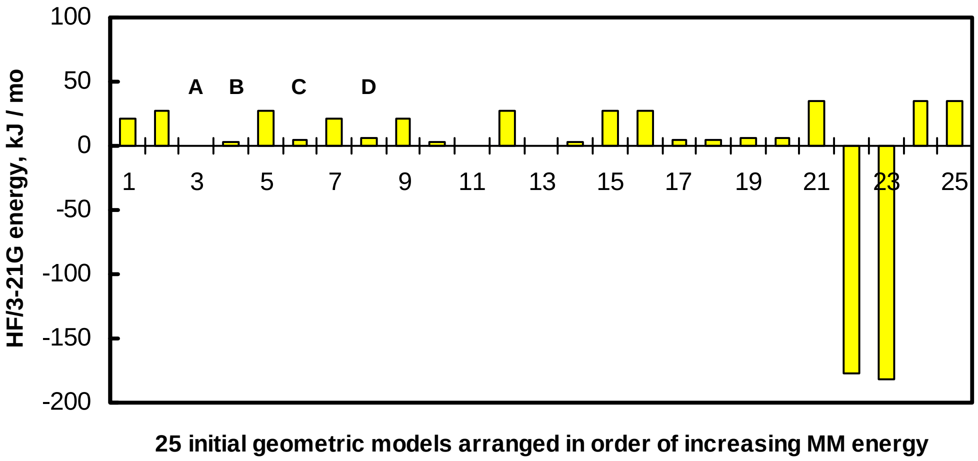

Figure S2. A graphical representation of transformations of 25 promising OPLS-AA models of *iso*-H_4**1**$^{4+}$ in the course of gas-phase *ab initio* HF/3-21G computations. Only four lowest-energy forms **A-D** of *iso*-H_4**1**$^{4+}$ were indicated.

Table S2. Key energetic, structural and NMR data for the forms **N1** and **N2** of the cation *n*-H_4**1**$^{4+}$ (used as 'reference' systems).[a]

Calculational result / Species	**N1** (gas)	**N2** (gas)	**N1**$_{hydr}$ [b]	**N2**$_{hydr}$ [b]
ΔE_{tot} [kJ mol^{-1}] [c]	0.00 [d]	0.54	(0.52)	(0.00) [e]
$\Delta G^{o}_{298.15}$ [kJ mol^{-1}] [f]	0.00 [d]	–0.66	(4.23)	(0.00) [e]
DFT-D3 correction [kJ mol^{-1}] [g]	–142.96	–142.16	–143.57	–143.70
vdW (DFT-D3) corrected $\Delta G^{o}_{298.15}$ [kJ mol^{-1}] [h]	0.00 [d]	0.14	(4.36)	(0.00) [e]
First vibrational mode ν_1 [cm^{-1}]	34.63	32.79	32.67	32.07
σ_C^{calc} for C11 [ppm] [i]	138.1473	136.3995	138.4700	137.7573
σ_C^{calc} for C12 [ppm] [i]	146.2779	145.6967	147.3211	147.2772
The referred form of *iso*-H_4**1**$^{4+}$	**A**, **C**	**B**, **D**	**A**$_{hydr}$, **C**$_{hydr}$	**B**$_{hydr}$, **D**$_{hydr}$

[a] For the *in vacuo* (or 'in H_2O solution', by using a new IEF-PCM solvation approach) DFT-B3LYP/6-31G(*d*)-optimized structures. [b] See Figs. S3 and S4, respectively. [c] Relative changes in classical (raw) total electronic energies E_{tot}. [d] The absolute value: E_{tot} / G^{o}_{298} / vdW corrected G^{o}_{298} of –749.3946402 / –748.967686/ –749.022135 hartrees; 1 Ha = 1 au = 2625.50 kJ mol^{-1}. [e] Absolute PCM/H_2O data: E_{tot}, G^{o}_{298}, and approximate vdW corrected G^{o}_{298} of –750.2594815, –749.824411, and –749.879145 Ha, respectively. [f] Relative changes in the standard Gibbs free energy. [g] See also Tables S12-S15. [h] See, the main text. [i] The GIAO-derived isotropic magnetic shielding tensor (see Methods in the main text).

Table S3. Important energetic, structural, and NMR data computed for forms **A-D** of *iso*-H_4**1**$^{4+}$.[a]

Calculational result / Species	**A** (gas)	**B** (gas)	**C** (gas)	**D** (gas)	$\mathbf{A}_{\mathbf{hydr}}$ [b]	$\mathbf{B}_{\mathbf{hydr}}$	$\mathbf{C}_{\mathbf{hydr}}$	$\mathbf{D}_{\mathbf{hydr}}$ [b]
ΔE_{tot} [kJ mol^{-1}] [c]	0.00 [d]	2.47	3.58	5.56	(7.26)	(7.31)	(8.06)	(0.00) [e]
$\Delta G^{o}_{298.15}$ [kJ mol^{-1}] [f]	0.00 [d]	1.81	2.99	4.38	(3.37)	(4.88)	(5.78)	(0.00) [e]
DFT-D3 correction [kJ mol^{-1}] [g,h]	–146.71	–146.45	–147.13	–146.38	–150.55	–149.86	–150.50	–148.54
vdW corrected ΔG^{o}_{298} [kJ mol^{-1}] [h]	0.00 [d]	2.06	2.57	4.71	(1.36)	(3.57)	(3.83)	(0.00) [e]
First vibrational mode ν_1 [cm^{-1}]	36.94	34.25	33.41	31.53	36.69	40.24	47.29	45.24
N^1–C11 [Å]	1.5872	1.5859	1.5828	1.5854	1.5293	1.5328	1.5283	1.5278
C12–N^5 [Å]	1.4422	1.4431	1.4429	1.4430	1.4626	1.4619	1.4630	1.4630
Σ ∠ around the atom N^5 [°] [i]	336.97	336.24	336.10	335.81	325.39	325.19	325.12	325.00
σ_C^{calc} for C11 [ppm] [j]	122.6781	121.6813	123.7106	122.5444	130.4028	127.9645	131.1863	131.1934
σ_C^{calc} for C12 [ppm] [j]	148.4401	148.7409	149.4372	148.7246	149.3172	149.1869	150.4476	150.5231
The 'reference' form of *n*-$H_4$1^{4+}	N1	N2	N1	N2	$N1_{hydr}$	$N2_{hydr}$	$N1_{hydr}$	$N2_{hydr}$
$\Delta\delta_C^{calc}$ for C11 [ppm] [k]	15.469	14.718	14.437	13.855	8.067	9.793	7.284	6.564
$\Delta\delta_C^{calc}$ for C12 [ppm] [k]	–2.162	–3.044	–3.159	–3.028	–1.996	–1.910	–3.126	–3.246

[a] For the *in vacuo* (or 'in H_2O solution', by using the IEF-PCM solvation method) B3LYP/6-31G(*d*)-optimized structures. [b] See Fig. 2 in the main text. [c] Relative changes in classical (raw) total electronic energies E_{tot} [d] Absolute E_{tot} / G^{o}_{298} / vdW (DFT-D3) corrected G^{o}_{298} of –749.320293 / –748.894423 / –748.950301 hartrees. [e] PCM/H_2O data: E_{tot}, G^{o}_{298}, and vdW corrected $G^{o}_{298.15}$ of –750.239890, –749.801617, and –749.858194 Ha, respectively. [f] Relative changes in the Gibbs free energy. [g] See also Tables S4-S11. [h] That is an original ΔG^{o}_{298} data + DFT-D3 correction; see, the main text. [i] The sum of three valence angles around the N^5 atom. [j] A GIAO-calculated shielding constant; see the main text (Methods). [k] The difference in σ_Cs due to a rearrangement *n*-H_4**1**$^{4+}$ → *iso*-H_4**1**$^{4+}$, $\Delta\delta_{Ci}^{calc} = \sigma_{Ci}^{calc}(\text{ref}) - \sigma_{Ci}^{calc}(\text{C11/C12})$, where σ_{Ci}^{calc}(ref)s for the reference forms **N1-N2**$_{\mathbf{hydr}}$ of *n*-H_4**1**$^{4+}$ were taken from Table S2.

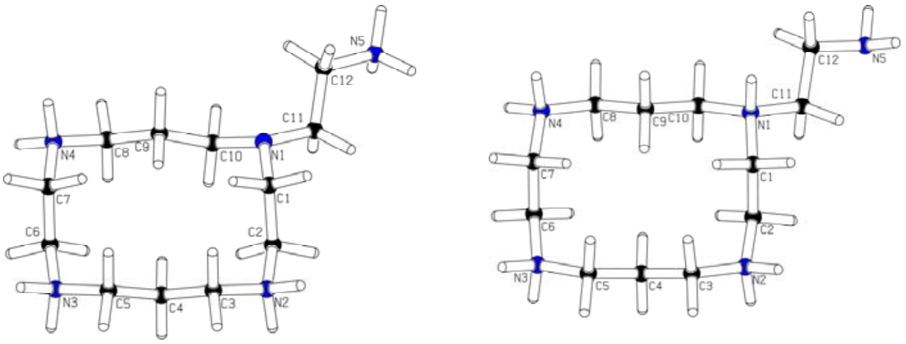

Figure S3. The PLATON views of the PCM/H_2O B3LYP/6-31G(*d*)-optimized conformer $\mathbf{N1}_{hydr}$ of *n*-$H_4\mathbf{1}^{4+}$ (left side) being in a postulated prototropic equilibrium with the form $\mathbf{A}_{hydr}$ of *iso*-$H_4\mathbf{1}^{4+}$ (right side); the N atoms are in blue.

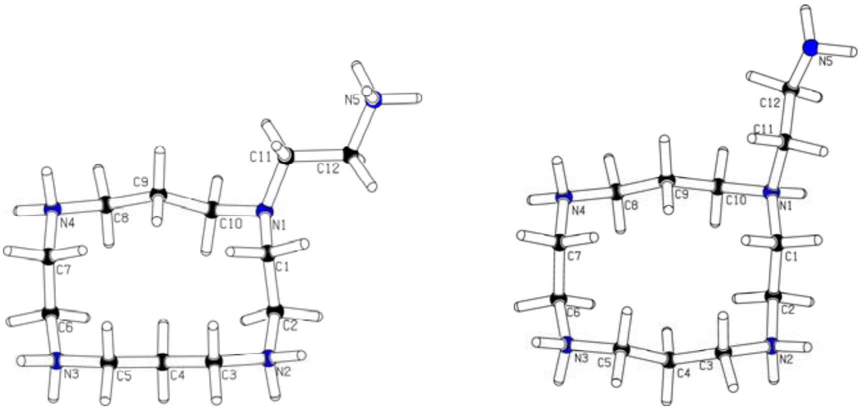

Figure S4. The PLATON view of the PCM/H_2O B3LYP/6-31G(*d*)-optimized conformer $\mathbf{N2}_{hydr}$ of *n*-$H_4\mathbf{1}^{4+}$ (left side) being in a postulated prototropic equilibrium with the form $\mathbf{D}_{hydr}$ of *iso*-$H_4\mathbf{1}^{4+}$ (right side); the N atoms are in blue.

Correction of an earlier explanation

given in the main text, Table S1, and Fig. S1 in Ref. [6]:

Nazarski, R.B.: Physical image versus structure relation. Part 14–An attempt to rationalize some acidic region ^{13}C NMR-pH titration shifts for tetraaza macrocycles throughout the conformational GIAO DFT computational results: a pendant-arm cyclam case. *J. Phys. Org. Chem.* **22**, 834-844 (2009).

Substantial broadening of the ^{13}C NMR signals coming from the atoms C3–C7 (see Fig. S5) results evidently from a conformational change involving five macrocyclic ring CH_2 groups in the close neighborhood of the protonated atom N^3, which is associated with a dynamic equilibrium:

$$H_3\mathbf{1}^{3+} + H^+ \rightleftharpoons H_4\mathbf{1}^{4+}$$

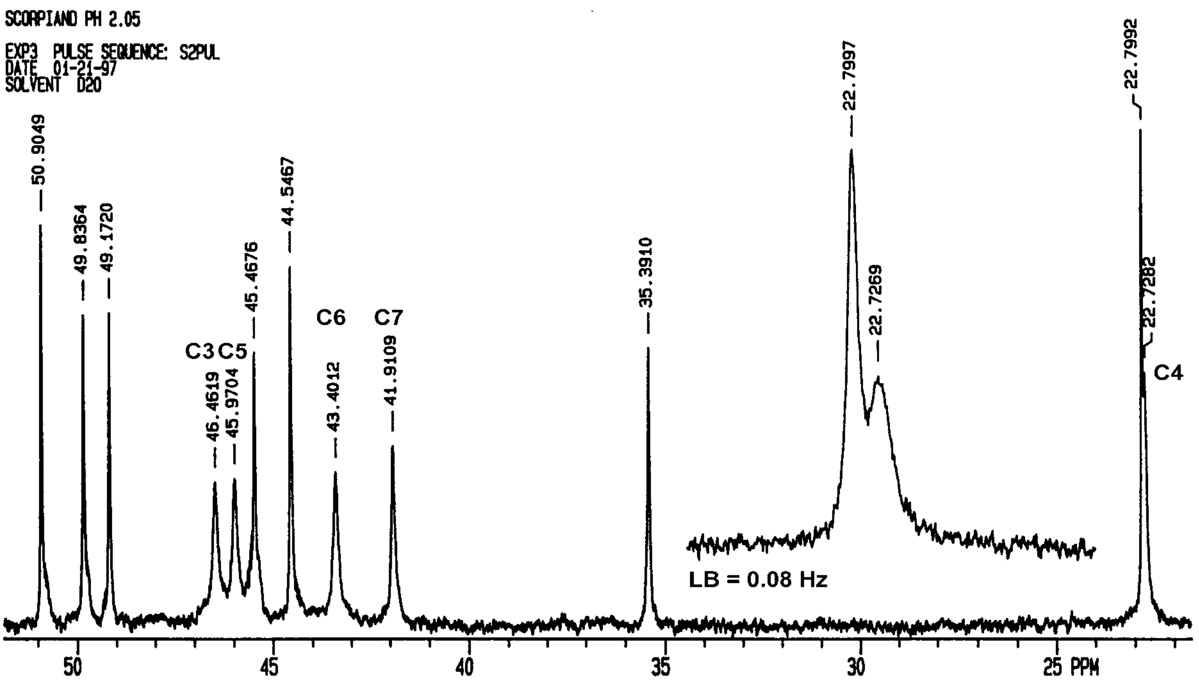

Figure S5. The 50.29 MHz $^{13}C\{^1H\}$ NMR spectrum of amine **1** in H_2O/D_2O (~95:5 v/v) registered at pH 2.05 (10900 transients, the 0.65/0.08 Hz line broadening; for other NMR measurement details, see Ref. [6]). Significantly broadened signals are due to the macrocyclic ring carbons C3–C7; for an arbitrary atom numbering applied here, see structural formula given in Fig. S1.

Table S4. Cartesian coordinates for the conformer A of *iso*-$H_4 1^{4+}$ (*iso*-$H_4 1^{4+}$A)

[*In vacuo* B3LYP/6-31G(d) model, N_{imag} = 0, E(RB3LYP) = -749.320292746 Ha]

Center Number	Atomic Number	Atomic Type	Coordinates (Angstroms) X	Y	Z
1	7	0	2.204371	-0.091827	0.546880
2	6	0	1.464803	1.201381	0.853030
3	6	0	1.073173	1.996235	-0.410868
4	7	0	0.076383	3.120241	-0.116463
5	6	0	-1.315529	2.786904	0.440313
6	6	0	-2.112636	1.798649	-0.439077
7	6	0	-3.414178	1.383240	0.280220
8	7	0	-4.190357	0.269442	-0.443670
9	6	0	-3.503143	-1.060064	-0.754771
10	6	0	-3.083963	-1.831701	0.516465
11	7	0	-2.080075	-2.946823	0.228594
12	6	0	-0.686681	-2.599193	-0.328153
13	6	0	0.102433	-1.607941	0.551649
14	6	0	1.406704	-1.179070	-0.164028
15	6	0	3.593683	0.157801	-0.178901
16	6	0	4.669842	-0.930077	0.075352
17	7	0	5.829806	-0.402698	-0.600218
18	1	0	2.472650	-0.465914	1.468480
19	1	0	-0.034322	3.656200	-0.992143
20	1	0	0.507200	3.797580	0.534113
21	1	0	-4.539921	0.638553	-1.343084
22	1	0	-5.051470	0.094717	0.100908
23	1	0	-1.962489	-3.471816	1.110411
24	1	0	-2.503672	-3.636703	-0.413898
25	1	0	6.221781	-1.028109	-1.296705
26	1	0	6.569079	-0.099819	0.026553
27	1	0	2.140370	1.789304	1.481851
28	1	0	0.595280	0.946022	1.463005
29	1	0	1.942243	2.488144	-0.856971
30	1	0	0.613777	1.375824	-1.181830
31	1	0	-1.825844	3.751673	0.524310
32	1	0	-1.160666	2.408286	1.453696
33	1	0	-2.346676	2.258634	-1.407527
34	1	0	-1.501420	0.910739	-0.635146
35	1	0	-4.120231	2.217003	0.349406
36	1	0	-3.232323	1.023528	1.296201
37	1	0	-4.226297	-1.634180	-1.343611
38	1	0	-2.660043	-0.822667	-1.405258
39	1	0	-3.945709	-2.322327	0.981161
40	1	0	-2.620835	-1.191688	1.268932
41	1	0	-0.173310	-3.562159	-0.413081
42	1	0	-0.845153	-2.223043	-1.342166
43	1	0	0.336708	-2.068016	1.520061
44	1	0	-0.516673	-0.726317	0.751122
45	1	0	1.213418	-0.814457	-1.176611
46	1	0	2.091701	-2.025578	-0.254985
47	1	0	3.954805	1.110675	0.213937
48	1	0	3.377056	0.254603	-1.245031
49	1	0	4.817066	-1.068944	1.156815
50	1	0	4.365970	-1.895070	-0.346157

Van der Waals correction (DFT-D V3) -0.055877867 Ha

Table S5. Cartesian coordinates for the conformer B of *iso*-H_41^{4+} (*iso*-H_41^{4+}B)

[*In vacuo* B3LYP/6-31G(d) model, N_{imag} = 0, E(RB3LYP) = -749.319351803 Ha]

Center Number	Atomic Number	Atomic Type	Coordinates (Angstroms) X	Y	Z
1	7	0	2.247538	-0.040017	0.657363
2	6	0	1.666960	1.225356	0.053026
3	6	0	0.879609	2.097843	1.057452
4	7	0	0.023511	3.169330	0.377139
5	6	0	-1.125385	2.752361	-0.553157
6	6	0	-2.154949	1.816170	0.116915
7	6	0	-3.175731	1.319107	-0.929505
8	7	0	-4.145940	0.255031	-0.387991
9	6	0	-3.599403	-1.027874	0.238266
10	6	0	-2.804521	-1.895069	-0.763570
11	7	0	-1.940415	-2.950772	-0.076391
12	6	0	-0.789520	-2.512429	0.847384
13	6	0	0.254185	-1.611077	0.155443
14	6	0	1.241719	-1.052360	1.204987
15	6	0	3.295739	-0.690537	-0.339162
16	6	0	4.696347	-0.026438	-0.321850
17	7	0	5.459837	-0.842726	-1.234634
18	1	0	2.821817	0.232638	1.468727
19	1	0	-0.355930	3.768529	1.127841
20	1	0	0.633623	3.805672	-0.161652
21	1	0	-4.756590	0.692332	0.321425
22	1	0	-4.794986	0.015704	-1.156140
23	1	0	-2.542937	-3.591181	0.466752
24	1	0	-1.555395	-3.550299	-0.824318
25	1	0	6.144548	-1.436133	-0.775546
26	1	0	5.923824	-0.314497	-1.966728
27	1	0	1.056554	0.909000	-0.794692
28	1	0	2.517227	1.782580	-0.349257
29	1	0	0.198358	1.522977	1.685955
30	1	0	1.556899	2.646034	1.720562
31	1	0	-0.661935	2.297234	-1.432019
32	1	0	-1.585783	3.692420	-0.873484
33	1	0	-1.634815	0.958785	0.558309
34	1	0	-2.672505	2.342985	0.928676
35	1	0	-2.692957	0.880075	-1.806358
36	1	0	-3.819707	2.131161	-1.282107
37	1	0	-3.003854	-0.717458	1.098078
38	1	0	-4.474734	-1.565946	0.617180
39	1	0	-2.129982	-1.309907	-1.390318
40	1	0	-3.478307	-2.448776	-1.425848
41	1	0	-1.255036	-2.030044	1.710773
42	1	0	-0.342860	-3.447915	1.198925
43	1	0	-0.251980	-0.781127	-0.351315
44	1	0	0.795012	-2.179084	-0.610161
45	1	0	1.847522	-1.854138	1.636337
46	1	0	0.711900	-0.574317	2.034798
47	1	0	2.851572	-0.650421	-1.335691
48	1	0	3.386929	-1.730447	-0.020114
49	1	0	4.647186	1.012770	-0.668294
50	1	0	5.095300	-0.015436	0.703922

Van der Waals correction (DFT-D V3)	-0.055780781 Ha

Table S6. Cartesian coordinates for the conformer C of *iso*-$H_4 1^{4+}$ (*iso*-$H_4 1^{4+}$C)

[*In vacuo* B3LYP/6-31G(d) model, N_{imag} = 0, E(RB3LYP) = -749.318928637 Ha]

Center Number	Atomic Number	Atomic Type	Coordinates (Angstroms) X	Y	Z
1	7	0	2.169104	-0.632816	0.391327
2	6	0	1.684256	0.765925	0.741157
3	6	0	1.330607	1.626786	-0.491420
4	7	0	0.529026	2.880735	-0.128648
5	6	0	-0.874313	2.751326	0.480953
6	6	0	-1.861184	1.955948	-0.401184
7	6	0	-3.187178	1.735927	0.359372
8	7	0	-4.154780	0.774914	-0.351929
9	6	0	-3.700944	-0.643266	-0.697537
10	6	0	-3.363687	-1.485634	0.552857
11	7	0	-2.553124	-2.737462	0.223814
12	6	0	-1.142838	-2.601072	-0.379561
13	6	0	-0.187160	-1.728665	0.460435
14	6	0	1.159247	-1.557575	-0.284413
15	6	0	3.539252	-0.637215	-0.401048
16	6	0	4.767639	-0.177568	0.421942
17	7	0	5.865502	-0.355559	-0.497282
18	1	0	2.409156	-1.057731	1.298545
19	1	0	1.084681	3.466094	0.516312
20	1	0	0.466440	3.451311	-0.987041
21	1	0	-4.466984	1.209456	-1.235756
22	1	0	-5.016049	0.732654	0.218204
23	1	0	-3.098504	-3.348409	-0.406766
24	1	0	-2.487157	-3.283139	1.098374
25	1	0	6.405603	-1.199563	-0.329187
26	1	0	6.501182	0.435036	-0.533234
27	1	0	2.493442	1.229288	1.312024
28	1	0	0.834370	0.651092	1.417641
29	1	0	2.229521	1.998500	-0.990404
30	1	0	0.739436	1.090141	-1.234962
31	1	0	-1.212682	3.781292	0.632854
32	1	0	-0.743194	2.300222	1.467683
33	1	0	-2.049574	2.494329	-1.338602
34	1	0	-1.419269	0.987911	-0.661430
35	1	0	-3.748225	2.669599	0.468285
36	1	0	-3.030641	1.330233	1.362122
37	1	0	-4.527727	-1.087793	-1.261539
38	1	0	-2.855322	-0.536152	-1.378575
39	1	0	-4.274732	-1.843352	1.044062
40	1	0	-2.782235	-0.933765	1.292879
41	1	0	-0.778347	-3.629569	-0.466794
42	1	0	-1.276367	-2.215321	-1.393558
43	1	0	-0.018109	-2.191650	1.441140
44	1	0	-0.644509	-0.748470	0.635475
45	1	0	1.020066	-1.180446	-1.301533
46	1	0	1.668381	-2.521296	-0.369422
47	1	0	3.398493	-0.025037	-1.293826
48	1	0	3.679753	-1.674314	-0.712753
49	1	0	4.682271	0.876955	0.709018
50	1	0	4.844557	-0.766278	1.350454

Van der Waals correction (DFT-D V3) -0.056037582 Ha

Table S7. Cartesian coordinates for the conformer D of *iso*-$H_4 1^{4+}$ (*iso*-$H_4 1^{4+}$D)

[*In vacuo* B3LYP/6-31G(d) model, N_{imag} = 0, E(RB3LYP) = -749.318174565 Ha]

Center Number	Atomic Number	Atomic Type	Coordinates (Angstroms) X	Y	Z
1	7	0	2.288143	0.503300	0.446906
2	6	0	1.448955	1.637779	-0.116847
3	6	0	0.529485	2.316593	0.924943
4	7	0	-0.512345	3.241431	0.290135
5	6	0	-1.616524	2.644536	-0.594640
6	6	0	-2.473756	1.583689	0.129702
7	6	0	-3.434714	0.901718	-0.867215
8	7	0	-4.183926	-0.301684	-0.269246
9	6	0	-3.397319	-1.454671	0.354043
10	6	0	-2.490527	-2.181651	-0.663986
11	7	0	-1.428924	-3.053473	0.004591
12	6	0	-0.323495	-2.399503	0.854457
13	6	0	0.507163	-1.349583	0.087886
14	6	0	1.522775	-0.679697	1.043874
15	6	0	3.395649	0.046674	-0.591550
16	6	0	4.672550	-0.535109	0.065004
17	7	0	5.527015	-0.815359	-1.063502
18	1	0	2.839946	0.885046	1.229356
19	1	0	-0.033465	3.967432	-0.267855
20	1	0	-0.951977	3.771910	1.059488
21	1	0	-4.834278	0.038872	0.457785
22	1	0	-4.809766	-0.668060	-1.005911
23	1	0	-0.984225	-3.604286	-0.747526
24	1	0	-1.887769	-3.767689	0.594144
25	1	0	5.955729	-1.734993	-1.030613
26	1	0	6.257084	-0.122659	-1.203158
27	1	0	0.899322	1.226457	-0.965865
28	1	0	2.160261	2.367105	-0.514207
29	1	0	-0.028124	1.607759	1.537932
30	1	0	1.108876	2.953401	1.601544
31	1	0	-1.119626	2.242899	-1.481297
32	1	0	-2.219955	3.500296	-0.913673
33	1	0	-1.816980	0.829939	0.577415
34	1	0	-3.043233	2.049969	0.943667
35	1	0	-2.919747	0.533692	-1.758287
36	1	0	-4.221901	1.584676	-1.202296
37	1	0	-2.841380	-1.028772	1.190733
38	1	0	-4.149855	-2.134585	0.767215
39	1	0	-1.951903	-1.496108	-1.319847
40	1	0	-3.074339	-2.858614	-1.296482
41	1	0	-0.816797	-1.982547	1.736215
42	1	0	0.296647	-3.235853	1.192461
43	1	0	-0.161344	-0.590126	-0.333462
44	1	0	1.028961	-1.824855	-0.750207
45	1	0	2.291997	-1.391529	1.352842
46	1	0	1.034330	-0.322715	1.955667
47	1	0	3.654663	0.942668	-1.158956
48	1	0	2.928167	-0.673070	-1.264377
49	1	0	5.088991	0.186597	0.786103
50	1	0	4.450498	-1.456406	0.615242

Van der Waals correction (DFT-D V3)	-0.055752392 Ha

Table S8. Cartesian coordinates for the conformer A_{hydr} of *iso*-H_4**1**$^{4+}$ (*iso*-H_4**1**$^{4+}$$A_{hydr}$)

[B3LYP/6-31G(d)/PCM(H_2O) model, N_{imag} = 0, E(RB3LYP) = -750.237124084 Ha]

Center Number	Atomic Number	Atomic Type	Coordinates (Angstroms) X	Y	Z
1	7	0	2.181141	-0.059944	0.554321
2	6	0	1.395124	1.207563	0.848381
3	6	0	1.010253	1.975098	-0.423576
4	7	0	0.034237	3.098151	-0.134803
5	6	0	-1.336400	2.767465	0.440059
6	6	0	-2.100073	1.758896	-0.420319
7	6	0	-3.391677	1.336940	0.283284
8	7	0	-4.112973	0.218296	-0.457228
9	6	0	-3.367204	-1.063643	-0.762116
10	6	0	-2.985920	-1.816935	0.517891
11	7	0	-2.003445	-2.937634	0.249501
12	6	0	-0.629717	-2.600933	-0.314356
13	6	0	0.120351	-1.576216	0.540402
14	6	0	1.415668	-1.164379	-0.166981
15	6	0	3.509680	0.243197	-0.139741
16	6	0	4.516032	-0.909702	-0.045321
17	7	0	5.784033	-0.401692	-0.567986
18	1	0	2.429057	-0.427896	1.481894
19	1	0	-0.092290	3.610308	-1.016660
20	1	0	0.475699	3.780203	0.495481
21	1	0	-4.450894	0.585485	-1.355778
22	1	0	-4.967498	-0.002429	0.069043
23	1	0	-1.882553	-3.442444	1.136364
24	1	0	-2.433685	-3.626819	-0.380957
25	1	0	6.386717	-1.184784	-0.810078
26	1	0	6.274372	0.119312	0.157202
27	1	0	2.036447	1.814510	1.489504
28	1	0	0.530318	0.915070	1.441472
29	1	0	1.876627	2.459798	-0.874088
30	1	0	0.550937	1.345486	-1.184030
31	1	0	-1.857652	3.723470	0.503533
32	1	0	-1.174546	2.404985	1.455940
33	1	0	-2.323418	2.182676	-1.404554
34	1	0	-1.473134	0.880736	-0.578662
35	1	0	-4.111184	2.155624	0.332789
36	1	0	-3.212908	0.985136	1.299760
37	1	0	-4.041543	-1.660039	-1.378807
38	1	0	-2.509913	-0.792145	-1.375523
39	1	0	-3.860369	-2.293008	0.964819
40	1	0	-2.535244	-1.177078	1.274843
41	1	0	-0.100516	-3.553388	-0.363548
42	1	0	-0.785174	-2.249463	-1.334951
43	1	0	0.338922	-1.987822	1.530894
44	1	0	-0.513306	-0.701027	0.682891
45	1	0	1.230894	-0.811700	-1.182727
46	1	0	2.101906	-2.008646	-0.226153
47	1	0	3.913506	1.125411	0.361335
48	1	0	3.296063	0.488886	-1.180280
49	1	0	4.563856	-1.277397	0.992972
50	1	0	4.202852	-1.745356	-0.676221

Van der Waals correction (DFT-D V3)   -0.057343268 Ha

Table S9. Cartesian coordinates for the conformer B_{hydr} of *iso*-$H_4 1^{4+}$ (*iso*-$H_4 1^{4+} B_{hydr}$)

[B3LYP/6-31G(d)/PCM(H_2O) model, N_{imag} = 0, E(RB3LYP) = -750.237104706 Ha]

Center Number	Atomic Number	Atomic Type	Coordinates (Angstroms) X	Y	Z
1	7	0	2.219292	-0.070039	0.627668
2	6	0	1.611032	1.180705	0.021965
3	6	0	0.861779	2.039661	1.049550
4	7	0	0.037001	3.122116	0.384129
5	6	0	-1.090585	2.720431	-0.556467
6	6	0	-2.117175	1.809373	0.119028
7	6	0	-3.135745	1.316849	-0.912303
8	7	0	-4.085868	0.276752	-0.335435
9	6	0	-3.512614	-0.990350	0.264020
10	6	0	-2.762416	-1.832961	-0.774668
11	7	0	-1.927414	-2.915292	-0.123586
12	6	0	-0.797740	-2.514180	0.814852
13	6	0	0.230350	-1.603994	0.137930
14	6	0	1.223185	-1.085609	1.181604
15	6	0	3.216681	-0.702526	-0.349452
16	6	0	4.585803	-0.012701	-0.304727
17	7	0	5.465234	-0.745902	-1.213632
18	1	0	2.775449	0.217271	1.442731
19	1	0	-0.352175	3.702566	1.137839
20	1	0	0.659452	3.755461	-0.133786
21	1	0	-4.649565	0.722026	0.399306
22	1	0	-4.764017	0.031378	-1.068351
23	1	0	-2.544241	-3.555424	0.392849
24	1	0	-1.538994	-3.487966	-0.883583
25	1	0	5.833271	-1.572662	-0.745771
26	1	0	6.271590	-0.168439	-1.440607
27	1	0	0.977706	0.852780	-0.800094
28	1	0	2.437875	1.745737	-0.407889
29	1	0	0.181400	1.470730	1.680570
30	1	0	1.561821	2.562728	1.703473
31	1	0	-0.626432	2.250728	-1.425228
32	1	0	-1.538808	3.660223	-0.882055
33	1	0	-1.604620	0.952224	0.558454
34	1	0	-2.623130	2.338959	0.932650
35	1	0	-2.657753	0.865491	-1.782617
36	1	0	-3.779371	2.125584	-1.261942
37	1	0	-2.888407	-0.684773	1.101681
38	1	0	-4.364566	-1.538102	0.669546
39	1	0	-2.090651	-1.248106	-1.400359
40	1	0	-3.460145	-2.352579	-1.433284
41	1	0	-1.260977	-2.046092	1.685008
42	1	0	-0.349590	-3.454249	1.139858
43	1	0	-0.283946	-0.761155	-0.327071
44	1	0	0.749490	-2.147253	-0.656850
45	1	0	1.834203	-1.891587	1.590392
46	1	0	0.714219	-0.607364	2.019162
47	1	0	2.784193	-0.657746	-1.349331
48	1	0	3.314519	-1.747838	-0.051560
49	1	0	4.507540	1.017983	-0.663473
50	1	0	4.941613	0.027844	0.738338

Van der Waals correction (DFT-D V3)	-0.057077907 Ha

Table S10. Cartesian coordinates for the conformer C_{hydr} of *iso*-$H_4 1^{4+}$ (*iso*-$H_4 1^{4+} C_{hydr}$)

[B3LYP/6-31G(d)/PCM(H_2O) model, N_{imag} = 0, E(RB3LYP) = -750.236820681 Ha]

Center Number	Atomic Number	Atomic Type	Coordinates (Angstroms) X	Y	Z
1	7	0	2.145495	-0.659405	0.356373
2	6	0	1.632483	0.727113	0.709311
3	6	0	1.276002	1.570188	-0.521340
4	7	0	0.522157	2.829610	-0.142066
5	6	0	-0.854207	2.714699	0.498295
6	6	0	-1.847587	1.954934	-0.385399
7	6	0	-3.160878	1.730187	0.369806
8	7	0	-4.087919	0.770462	-0.362407
9	6	0	-3.599682	-0.627649	-0.676963
10	6	0	-3.267481	-1.428405	0.586891
11	7	0	-2.511120	-2.699524	0.261698
12	6	0	-1.137101	-2.598793	-0.386814
13	6	0	-0.165960	-1.741529	0.429975
14	6	0	1.148392	-1.580823	-0.338076
15	6	0	3.462964	-0.640792	-0.417995
16	6	0	4.636676	-0.079410	0.391717
17	7	0	5.838536	-0.281746	-0.417596
18	1	0	2.353452	-1.097118	1.262911
19	1	0	1.106318	3.397808	0.483471
20	1	0	0.436967	3.390759	-0.999397
21	1	0	-4.357960	1.197195	-1.258013
22	1	0	-4.964279	0.714434	0.172563
23	1	0	-3.092408	-3.299742	-0.336821
24	1	0	-2.419984	-3.221593	1.141891
25	1	0	6.172670	-1.237670	-0.305426
26	1	0	6.582505	0.319233	-0.070288
27	1	0	2.419278	1.205764	1.292474
28	1	0	0.785428	0.584577	1.378174
29	1	0	2.168814	1.919623	-1.039591
30	1	0	0.659672	1.040111	-1.245388
31	1	0	-1.173307	3.744335	0.669076
32	1	0	-0.715400	2.241838	1.471117
33	1	0	-2.034384	2.506413	-1.312142
34	1	0	-1.422247	0.990933	-0.666527
35	1	0	-3.729984	2.655434	0.470947
36	1	0	-3.005181	1.321026	1.368522
37	1	0	-4.406192	-1.102068	-1.238054
38	1	0	-2.750351	-0.521259	-1.349410
39	1	0	-4.175892	-1.745920	1.100762
40	1	0	-2.659445	-0.877946	1.302205
41	1	0	-0.789668	-3.629288	-0.477077
42	1	0	-1.286575	-2.204761	-1.392957
43	1	0	0.015996	-2.194233	1.410396
44	1	0	-0.611981	-0.760420	0.596117
45	1	0	0.993978	-1.186593	-1.343517
46	1	0	1.660417	-2.539463	-0.429009
47	1	0	3.300767	-0.079153	-1.338440
48	1	0	3.660895	-1.680506	-0.684375
49	1	0	4.517643	0.995272	0.554448
50	1	0	4.668659	-0.561121	1.382900

Van der Waals correction (DFT-D V3) -0.057322892 Ha

Table S11. Cartesian coordinates for the conformer D_{hydr} of *iso*-$H_4 1^{4+}$ (*iso*-$H_4 1^{4+} D_{hydr}$)

[B3LYP/6-31G(d)/PCM(H_2O) model, N_{imag} = 0, E(RB3LYP) = -750.239889748 Ha]

Center Number	Atomic Number	Atomic Type	Coordinates (Angstroms) X	Y	Z
1	7	0	2.226681	0.640965	0.467501
2	6	0	1.286870	1.699966	-0.075009
3	6	0	0.358935	2.295288	0.993359
4	7	0	-0.677560	3.209004	0.372583
5	6	0	-1.690918	2.608721	-0.591190
6	6	0	-2.615695	1.591385	0.087248
7	6	0	-3.303802	0.736706	-0.983119
8	7	0	-4.017380	-0.476388	-0.404234
9	6	0	-3.197977	-1.523864	0.316245
10	6	0	-2.240921	-2.249590	-0.637946
11	7	0	-1.218253	-3.071949	0.115185
12	6	0	-0.177815	-2.348547	0.956605
13	6	0	0.731699	-1.428791	0.132943
14	6	0	1.553920	-0.570314	1.105492
15	6	0	3.242083	0.266121	-0.610784
16	6	0	4.395491	-0.589911	-0.079128
17	7	0	5.373405	-0.694286	-1.162271
18	1	0	2.769468	1.077792	1.222555
19	1	0	-0.203771	3.982354	-0.111213
20	1	0	-1.183060	3.660509	1.145009
21	1	0	-4.740886	-0.154628	0.250626
22	1	0	-4.542214	-0.924387	-1.166251
23	1	0	-0.735068	-3.660904	-0.574500
24	1	0	-1.704277	-3.738289	0.728196
25	1	0	6.014344	-1.458958	-0.962527
26	1	0	5.943497	0.149635	-1.193942
27	1	0	0.742047	1.238629	-0.896400
28	1	0	1.926746	2.479711	-0.490432
29	1	0	-0.187884	1.545917	1.560924
30	1	0	0.914961	2.912865	1.700978
31	1	0	-1.122721	2.169939	-1.410914
32	1	0	-2.249132	3.457514	-0.988542
33	1	0	-2.037050	0.955654	0.759693
34	1	0	-3.360642	2.110411	0.698380
35	1	0	-2.599791	0.360518	-1.725581
36	1	0	-4.077638	1.298722	-1.508368
37	1	0	-2.687701	-1.017448	1.132678
38	1	0	-3.917784	-2.221231	0.748397
39	1	0	-1.684989	-1.576131	-1.287438
40	1	0	-2.783508	-2.952446	-1.271852
41	1	0	-0.722647	-1.804219	1.727815
42	1	0	0.393779	-3.139423	1.445300
43	1	0	0.132535	-0.792903	-0.522259
44	1	0	1.384462	-2.028704	-0.506643
45	1	0	2.355518	-1.147014	1.567035
46	1	0	0.933905	-0.186602	1.915263
47	1	0	3.630193	1.211513	-0.994344
48	1	0	2.701011	-0.236880	-1.412523
49	1	0	4.789287	-0.146897	0.850529
50	1	0	4.049535	-1.599065	0.160701

Van der Waals correction (DFT-D V3) -0.056577231 Ha

Table S12. Cartesian coordinates for the conformer N1 of *n*-$H_4 1^{4+}$ (*n*-$H_4 1^{4+}$N1)

[*In vacuo* B3LYP/6-31G(d) model, N_{imag} = 0, E(RB3LYP) = -749.394640202 Ha]

Center Number	Atomic Number	Atomic Type	Coordinates (Angstroms) X	Y	Z
1	7	0	-2.152678	-0.007063	-0.069327
2	6	0	-1.470966	1.216513	-0.482448
3	6	0	-0.899836	2.081042	0.679868
4	7	0	0.084344	3.168844	0.221966
5	6	0	1.367441	2.762799	-0.503628
6	6	0	2.230697	1.763009	0.293785
7	6	0	3.407438	1.278248	-0.575154
8	7	0	4.240032	0.167294	0.086394
9	6	0	3.554926	-1.120174	0.538792
10	6	0	2.952919	-1.919196	-0.636183
11	7	0	1.972255	-2.988907	-0.172748
12	6	0	0.678563	-2.564355	0.553937
13	6	0	-0.195943	-1.595079	-0.253861
14	6	0	-1.357124	-1.036584	0.614275
15	6	0	-3.553794	0.119821	0.376032
16	6	0	-4.491142	-0.671682	-0.567249
17	7	0	-5.957132	-0.582099	-0.149079
18	1	0	0.331071	3.733138	1.048685
19	1	0	-0.411465	3.828753	-0.396254
20	1	0	4.713191	0.558736	0.916266
21	1	0	5.015064	-0.060690	-0.557207
22	1	0	1.711177	-3.531443	-1.010632
23	1	0	2.460613	-3.671900	0.427684
24	1	0	-6.554286	-1.122908	-0.794071
25	1	0	-6.319380	0.382447	-0.161000
26	1	0	-2.171544	1.817897	-1.070519
27	1	0	-0.665650	0.947625	-1.175042
28	1	0	-1.690701	2.621077	1.207830
29	1	0	-0.364693	1.482813	1.418876
30	1	0	1.905856	3.696832	-0.690884
31	1	0	1.060685	2.356335	-1.469860
32	1	0	2.606623	2.234472	1.210724
33	1	0	1.612426	0.909164	0.588800
34	1	0	4.122612	2.082276	-0.775053
35	1	0	3.076877	0.884750	-1.539618
36	1	0	4.326273	-1.699049	1.057077
37	1	0	2.806968	-0.825147	1.275701
38	1	0	3.733036	-2.446523	-1.194690
39	1	0	2.405009	-1.289178	-1.337781
40	1	0	0.161622	-3.506053	0.761500
41	1	0	0.991099	-2.146919	1.514716
42	1	0	-0.607576	-2.087658	-1.142120
43	1	0	0.416682	-0.758378	-0.610062
44	1	0	-0.951437	-0.683127	1.579611
45	1	0	-2.030871	-1.862152	0.862169
46	1	0	-3.844021	1.176385	0.369415
47	1	0	-3.672207	-0.228487	1.411392
48	1	0	-4.440243	-0.289957	-1.589073
49	1	0	-4.248620	-1.736661	-0.580042
50	1	0	-6.131005	-0.959121	0.794021

Van der Waals correction (DFT-D V3) -0.054449502 Ha

Table S13. Cartesian coordinates for the conformer N2 of *n*-$H_4 1^{4+}$ (*n*-$H_4 1^{4+}$N2)

[*In vacuo* B3LYP/6-31G(d) model, N_{imag} = 0, E(RB3LYP) = -749.394435258 Ha]

Center Number	Atomic Number	Atomic Type	Coordinates (Angstroms) X	Y	Z
1	7	0	2.151386	-0.014162	0.084844
2	6	0	1.495090	1.180272	-0.442444
3	6	0	0.949316	2.065939	0.703707
4	7	0	-0.015183	3.166587	0.238114
5	6	0	-1.305467	2.783557	-0.487959
6	6	0	-2.175502	1.779341	0.296158
7	6	0	-3.369145	1.335477	-0.571560
8	7	0	-4.229931	0.241837	0.082511
9	6	0	-3.577767	-1.063332	0.532185
10	6	0	-2.984067	-1.868867	-0.642536
11	7	0	-2.037768	-2.966963	-0.175561
12	6	0	-0.737607	-2.576160	0.561375
13	6	0	0.196840	-1.665340	-0.247348
14	6	0	1.298914	-1.048393	0.678228
15	6	0	3.396125	-0.454434	-0.569921
16	6	0	4.617264	-0.160666	0.336296
17	7	0	5.936872	-0.621361	-0.279634
18	1	0	-0.252230	3.735358	1.064515
19	1	0	0.491879	3.818192	-0.379626
20	1	0	-4.696257	0.641018	0.912540
21	1	0	-5.008048	0.035234	-0.564554
22	1	0	-2.549298	-3.636127	0.421269
23	1	0	-1.785877	-3.516309	-1.011724
24	1	0	5.968567	-1.637094	-0.449054
25	1	0	6.726726	-0.407701	0.349455
26	1	0	0.687586	0.935116	-1.152973
27	1	0	2.228614	1.757333	-1.012846
28	1	0	0.406481	1.489702	1.453732
29	1	0	1.760127	2.588215	1.217734
30	1	0	-1.006086	2.388194	-1.460981
31	1	0	-1.835553	3.725579	-0.658281
32	1	0	-1.568055	0.908401	0.563066
33	1	0	-2.533681	2.235142	1.228082
34	1	0	-3.053368	0.942651	-1.541242
35	1	0	-4.062771	2.160804	-0.760358
36	1	0	-2.829515	-0.789038	1.276703
37	1	0	-4.366134	-1.627628	1.040903
38	1	0	-2.411227	-1.247723	-1.332002
39	1	0	-3.771509	-2.370337	-1.214404
40	1	0	-1.051365	-2.121438	1.504941
41	1	0	-0.265179	-3.532398	0.805003
42	1	0	-0.375832	-0.847281	-0.701258
43	1	0	0.656816	-2.224042	-1.070614
44	1	0	1.948906	-1.846177	1.048651
45	1	0	0.811051	-0.639633	1.573581
46	1	0	3.516631	0.047335	-1.537403
47	1	0	3.344862	-1.528750	-0.785262
48	1	0	4.730513	0.909577	0.522309
49	1	0	4.535480	-0.673942	1.297044
50	1	0	6.146152	-0.158172	-1.175965

Van der Waals correction (DFT-D V3)	-0.054144717 Ha

Table S14. Cartesian coordinates for the conformer $N1_{hydr}$ of *n*-H_41^{4+} (*n*-$H_41^{4+}N1_{hydr}$)

[B3LYP/6-31G(d)/PCM(H_2O) model, N_{imag} = 0, E(RB3LYP) = -750.259284385 Ha]

Center Number	Atomic Number	Atomic Type	Coordinates (Angstroms) X	Y	Z
1	7	0	-2.124824	0.014857	-0.240201
2	6	0	-1.382812	1.207588	-0.636788
3	6	0	-0.851398	2.051693	0.545045
4	7	0	0.129981	3.131656	0.116766
5	6	0	1.430867	2.703270	-0.535778
6	6	0	2.313888	1.857095	0.388872
7	6	0	3.444294	1.228863	-0.433693
8	7	0	4.156044	0.100129	0.297991
9	6	0	3.372129	-1.144491	0.654003
10	6	0	2.877692	-1.885439	-0.595192
11	7	0	1.899472	-2.986432	-0.249226
12	6	0	0.585189	-2.611988	0.431114
13	6	0	-0.302597	-1.698603	-0.415764
14	6	0	-1.373342	-1.029311	0.466920
15	6	0	-3.459832	0.259760	0.309488
16	6	0	-4.464449	-0.636687	-0.423662
17	7	0	-5.853452	-0.497276	0.156263
18	1	0	0.344175	3.708494	0.938557
19	1	0	-0.344667	3.768323	-0.534225
20	1	0	4.548654	0.467459	1.173911
21	1	0	4.975462	-0.163876	-0.261915
22	1	0	1.686977	-3.482057	-1.123859
23	1	0	2.368194	-3.688345	0.336752
24	1	0	-6.526726	-1.080084	-0.352663
25	1	0	-6.194700	0.468949	0.105948
26	1	0	-2.038297	1.824713	-1.258780
27	1	0	-0.558665	0.897144	-1.283212
28	1	0	-1.661411	2.582193	1.048547
29	1	0	-0.338771	1.443424	1.288631
30	1	0	1.930553	3.627826	-0.829751
31	1	0	1.160660	2.163179	-1.442961
32	1	0	2.728491	2.473069	1.192705
33	1	0	1.713883	1.074252	0.857460
34	1	0	4.225165	1.954274	-0.665279
35	1	0	3.084714	0.812175	-1.374598
36	1	0	4.051772	-1.766957	1.238717
37	1	0	2.560514	-0.825567	1.304662
38	1	0	3.705473	-2.370410	-1.115430
39	1	0	2.370283	-1.232548	-1.302418
40	1	0	0.100424	-3.566823	0.642332
41	1	0	0.851243	-2.158316	1.386756
42	1	0	-0.781321	-2.263809	-1.221373
43	1	0	0.303162	-0.923450	-0.890882
44	1	0	-0.900691	-0.649811	1.389010
45	1	0	-2.090468	-1.789691	0.789472
46	1	0	-3.733131	1.307207	0.151003
47	1	0	-3.502316	0.079284	1.394121
48	1	0	-4.533739	-0.365051	-1.477104
49	1	0	-4.202911	-1.692538	-0.343483
50	1	0	-5.890302	-0.783685	1.140805

Van der Waals correction (DFT-D V3) -0.054684169 Ha

Table S15. Cartesian coordinates for the conformer N2$_{hydr}$ of *n*-$H_4 1^{4+}$ (*n*-$H_4 1^{4+}$N2$_{hydr}$)

[B3LYP/6-31G(d)/PCM(H_2O) model, N_{imag} = 0, E(RB3LYP) = -750.259481483 Ha]

Center Number	Atomic Number	Atomic Type	Coordinates (Angstroms) X	Y	Z
1	7	0	2.153835	-0.108713	0.393368
2	6	0	1.539206	1.078289	-0.203656
3	6	0	0.882095	1.949837	0.876646
4	7	0	0.033561	3.070217	0.297722
5	6	0	-1.176697	2.701986	-0.540254
6	6	0	-2.230622	1.917961	0.248337
7	6	0	-3.275490	1.362638	-0.725735
8	7	0	-4.167149	0.301397	-0.097860
9	6	0	-3.533066	-0.978405	0.405069
10	6	0	-2.861046	-1.764412	-0.726665
11	7	0	-2.027372	-2.906209	-0.194268
12	6	0	-0.808217	-2.568241	0.659415
13	6	0	0.263734	-1.778684	-0.090918
14	6	0	1.237409	-1.122191	0.924056
15	6	0	3.277507	-0.634655	-0.385226
16	6	0	4.574501	-0.000077	0.128136
17	7	0	5.765362	-0.454669	-0.684006
18	1	0	-0.273194	3.663544	1.077691
19	1	0	0.631932	3.677173	-0.274525
20	1	0	-4.669478	0.720423	0.694869
21	1	0	-4.902727	0.070695	-0.777747
22	1	0	-2.627419	-3.546785	0.339347
23	1	0	-1.714913	-3.454666	-1.004669
24	1	0	5.888805	-1.472596	-0.651051
25	1	0	6.633195	-0.034524	-0.334381
26	1	0	0.819561	0.819369	-0.994760
27	1	0	2.326601	1.663513	-0.687036
28	1	0	0.228990	1.382251	1.536913
29	1	0	1.640314	2.437079	1.491394
30	1	0	-0.805201	2.135556	-1.393624
31	1	0	-1.578421	3.647245	-0.909168
32	1	0	-1.755212	1.101886	0.793363
33	1	0	-2.709036	2.564911	0.989872
34	1	0	-2.818273	0.907558	-1.604969
35	1	0	-3.958553	2.141229	-1.069171
36	1	0	-2.841025	-0.694474	1.195319
37	1	0	-4.342420	-1.554151	0.857191
38	1	0	-2.194117	-1.150805	-1.328719
39	1	0	-3.604878	-2.210833	-1.388466
40	1	0	-1.183037	-2.025725	1.528422
41	1	0	-0.432207	-3.531646	1.007711
42	1	0	-0.197061	-0.996776	-0.700241
43	1	0	0.804701	-2.437161	-0.778592
44	1	0	1.847182	-1.893859	1.403089
45	1	0	0.651488	-0.666063	1.729518
46	1	0	3.161399	-0.452378	-1.464219
47	1	0	3.330687	-1.718862	-0.250375
48	1	0	4.553200	1.088208	0.056789
49	1	0	4.766472	-0.287158	1.162047
50	1	0	5.679098	-0.190626	-1.671732

Van der Waals correction (DFT-D V3)	-0.054733912 Ha
